# *tRF-27* competitively Binds to G3BPs and Activates MTORC1 to Enhance HER2 Positive Breast Cancer Trastuzumab Tolerance

**DOI:** 10.7150/ijbs.87415

**Published:** 2024-07-15

**Authors:** Yaozhou He, Yincheng Liu, Jue Gong, Fan Yang, Chunxiao Sun, Xueqi Yan, Ningjun Duan, Yijia Hua, Tianyu Zeng, Ziyi Fu, Yan Liang, Wei Li, Xiang Huang, Jinhai Tang, Yongmei Yin

**Affiliations:** 1 Department of Oncology, Jiangsu Province Hospital and Nanjing Medical University First Affiliated Hospital, Nanjing, China.; 2 Department of General Surgery, Jiangsu Province Hospital and Nanjing Medical University First Affiliated Hospital, Nanjing, China.

**Keywords:** Trastuzumab resistance, HER2-positive breast cancer, tRNA-derived fragment, MTORC1, G3BPs

## Abstract

About 20% of breast cancer patients are positive for HER2. The efficacy of current treatments is limited by primary and secondary resistance to trastuzumab. tRNA-derived fragments (tRFs) have shown crucial regulatory roles in various cancers. This study aimed to evaluate the role of *tRF-27* in regulating the resistance of HER2-positive breast cancer against trastuzumab. *tRF-27* was highly expressed in trastuzumab-resistant cells, and its expression level could predict the resistance to trastuzumab. High expression of* tRF-27* promoted the growth and proliferation of trastuzumab-exposed cells. RNA-pulldown assay and mass spectrometry were performed to identify Ras GTPase-activating protein-binding proteins 1 and 2 (G3BPs) (two proteins targeted by *tRF-27*); RNA-immunoprecipitation (RIP) to confirm their bindings; co-immunoprecipitation (co-IP) and RNA-pulldown assay to determine the binding domains between G3BPs and *tRF-27*.*tRF-27* bound to the nuclear transport factor 2 like domain(NTF2 domain) of G3BPs through a specific sequence. *tRF-27* relied on G3BPs and NTF2 domain to increase trastuzumab tolerance. *tRF-27* competed with lysosomal associated membrane protein 1(LAMP1) for NTF2 domain, thereby inhibiting lysosomal localization of G3BPs and tuberous sclerosis complex (TSC). Overexpression of *tRF-27* inhibited phosphorylation of TSCs and promoted the activation of mechanistic target of rapamycin complex 1(MTORC1) to enhance cell proliferation and entice the resistance of HER2-positive breast cancer against trastuzumab.

## Introduction

Approximately 20% of breast cancer patients are positive for HER2 1. Trastuzumab, a specific monoclonal antibody against HER2, has largely prolongated the survival of these patients over the past two decades 2. However, primary and secondary resistance still limits its therapeutic potential 3.

Trastuzumab mainly relies on antibody-dependent cell-mediated cytotoxicity (ADCC) to kill tumor cells 4. Trastuzumab can bind to HER2, a protein on the surface of tumor cells, to disturb the PI3K/AKT/mTOR signaling pathway, thereby inhibiting cell growth and proliferation 5. How to suppress trastuzumab resistance has always been a research hotspot 6. Previous clinical studies have found that the mTOR expression level is higher in trastuzumab-resistant patients than in trastuzumab-sensitive patients, making mTOR a candidate marker that may be exploited to regulate resistance to trastuzumab 7, 8. However, more sensitive biomarkers are needed to cope with this issue.

tRNA-derived fragments (tRFs) are a class of RNA fragments arising from rocedural separation of tRNA 9. Recent studies have shown that these proteins play key roles in cellular energy metabolism and translation. Stimulated by stress or drugs, tRNA is programmed to be cleaved by specific proteins such as Dicer, Angiogenin, and RNase Z 10. Depending on cleavage sites, these small fragments can be classified as tRNA halves or tRNA-derived fragments. tRFs can be divided into 5′-tRFs, 3′-tRFs, 1′-tRFs, and i-tRFs 11. Recent studies have found that a subset of tRFs can exert miRNA-like effects (such as binding to Argonaute protein), which confers them functions of ceRNAs 10. Other tRFs regulate various genetic processes in cancer cells. For example, *Hani Goodarzi* identified a novel class of tRFs derived from tRNA (Glu), tRNA (Asp), tRNA (Gly), and tRNA (Tyr) that, upon induction, suppressed the stability of multiple oncogenic transcripts in breast cancer cells by displacing their 3'-untranslated regions (UTRs) from the RNA-binding protein YBX1 12. *Zhangli Su* identified that methylation of tRFs regulated gene silencing and the unfolded protein response in bladder cancer 13. *Ling Pan* revealed that tRF-21 promoted AKT2/1-mediated phosphorylation of heteroribonucleoprotein L (hnRNP L), thereby enhancing the interaction of hnRNP L with dead box helicase 17 (DDX17) to form alternative splicing complexes. As hnRNP L-DDX17 is activated, pre-caspase 9 and mH2A1 mRNAs are preferentially spliced to form caspase 9b and mH2A1.2, hence promoting the malignant differentiation of PDAC cells 14. In previous studies, we have noticed the association of tRFs with the chemoresistance in cancer patients. However, no studies have analyzed the molecular mechanisms of tRFs in enhancing trastuzumab resistance in HER2+ breast cancer.

Ras GTPase-activating protein-binding proteins 1 and 2 (G3BPs) are a family of RNA-binding proteins that include G3BP1 and G3BP2 in mammals 15, 16. Researchers have summarized the function of G3BPs from three aspects: RNA stabilization, protein subcellular localization, and stress granules assembling. G3BPs are widely recognized as core components of stress granules (SGs) 17, 18. G3BPs have an unexpected lysosomal function in recruiting TSC2 when SGs are absent. G3BP1 and G3BP2 share a common domain architecture that can be divided to three major domains: a nuclear transport factor 2-like domain in the N-terminal, a central segment largely disordered, and an RNA binding domain (RBD) in the C-terminal. However, the role of G3BPs in breast cancer and trastuzumab resistance has not been studied 19, 20.

In this study, we first recognized* tRF-27* as a marker to predict trastuzumab resistance. We detected *tRF-27* expression in trastuzumab sensitive breast cancer cells and trastuzumab resistant breast cancer cells. We assessed the dysregulation of *tRF-27* (downregulation vs. overexpression) in regulating the proliferation and survival of cancer cells exposed to trastuzumab *in vitro*, and cancer cell survival *in vivo*. Then, we assessed the underlying molecular mechanisms. This study could offer novel mechanistic insights into the role of *tRF-27* and G3BPs in trastuzumab resistance 21-23.

## Materials and Methods

### Cell lines

Trastuzumab-sensitive cell lines SKBR3 and BT474 and trastuzumab-resistant cell line JIMT-1 were obtained from the American Type Culture Collection (ATCC, Manassas, VA, USA) 24. The normal breast epithelial cell lines HBL-100 and HEK293T were obtained from the Cell Bank of the Chinese Academy of Sciences (Shanghai, China). SKBR3 cells were cultured in RPMI-1640 (Multicell, USA), while JIMT-1, HBL-100, and HEK293T in DMEM (Gibco, UK) containing 10% fetal bovine serum (FBS, Gibco) 100 U/ml penicillin and 100 μg/ml streptomycin (Multicell, USA). The cells were cultured at 37 °C in 5% CO_2_, and the cell medium was replaced every 48h.

### Patient samples and processing

This study was approved by the Ethics Committee of the First Affiliated Hospital of Nanjing Medical University. The blood was sampled as reported in our previous study 21. Tumor samples were collected from the patients at the First Affiliated Hospital of Nanjing Medical University. All samples were obtained from surgically resected BRCA tissues and reviewed by experienced pathologists at the First Affiliated Hospital of Nanjing Medical University. Positive HER2 was considered if the HER-2:CEP17 ratio was greater than or equal to 2 through fluorescence *in situ* hybridization (FISH) 25. The Miller-Payne scores were scored according to the Breast Cancer NCCN guideline. The samples were collected, immediately frozen in liquid nitrogen, embedded in paraffin using a routine procedure, and stored at -80 °C until use. The study protocol was approved by the ethics committee of the First Affiliated Hospital of Nanjing Medical University.

### RNA and RNA sequencing

Total RNA was extracted from the cells using TRIzol reagent (Life Technologies, USA) and subjected to high-throughput sequencing. Total RNA was isolated from patient serum using Trizol LS (Life Technologies, USA). The quality and concentration of the isolated RNA were assessed using an Agilent 2100 Bioanalyzer (Thermo Fisher Scientific). The OD 260/280 absorbance ratios of all the samples were between 1.8 and 2.0. Finally, RNA was resuspended in RNase-free water and stored at -80 °C.

Illumina NextSeq 500 raw sequencing reads that passed the Illumina chastity filter were sequenced as reported previously 21. The sequencing reads were aligned to mature tRNAs on the entire genome using MINTbase 26. The data for high-throughput sequencing were deposited in Gene Expression Omnibus (GEO) under accession number GSE107473.

### Quantitative real-time PCR (qRT-PCR)

Total RNA was extracted using TRIzol reagent (Takara, Japan), and approximately 1000 ng of RNA was reverse-transcribed into cDNA using Primescript RT Reagent (Takara, Japan). qRT-PCR was performed using the following PCR primers 27:

*β-actin* forward, 5′-TCACCCACACTGTGCCCATCTACGA-3'; *β-actin* reverse, 5′-CAGCGGAACCGCTCATTGCCAATGG-3′; *G3BP1* forward, 5′- CGGGCGGGAATTTGTGAGA -3′; *G3BP1* reverse, 5′- TCTGTCCGTAGACTGCATCTG -3′; *G3BP2* forward, 5′- GTAGGGCGGGAGTTTGTGAG -3′; *G3BP2* reverse, 5′- CTGGGGCTTTCCACTAGCATC -3′.

Total RNA extracted from cells and BRCA samples was pretreated with an rtStar™ tRF&tiRNA Pretreatment Kit (Arraystar, USA) to remove terminal and internal methylations for efficient qRT-PCR. RNA was quantified by qRT-PCR using a Bulge-Loop miRNA qRT-PCR Stater Kit (Ribobio, China). PCR primers for tRFs were specifically designed and purchased from the manufacturer (Ribobio, China) 28.

### Lentivirus transfection and siRNA transfection

The lentiviruses used to knock down or overexpress *tRF-27* were purchased from Genechem (China). These lentiviruses were constructed based on the vector GV280. The carrier element order was: hU6-MCS-Ubiquitin-EGFP-IRES-puromycin. The lentiviruses were transfected into the cells according to the manufacturer's instructions. The siRNA of G3BP2 were specifically designed and purchased from Ribobio (China). Lipofectamine® 3000 transfection agent (Invitrogen) was used to transfect siRNA into the cells.

### Experimental proliferation and trastuzumab resistance assays

The cells were exposed to trastuzumab, with a concentration set at 2 μg/ml, which is similar to the clinical concentration 4. The Cell Counting Kit-8 (CCK-8) assay was used to detect cell survival and growth. Every 2*10^3 cells were cultured in one well of 96-well plates with 100μL of medium. Then, 10μL of CCK-8 was added to each well, and the absorbance was measured at 450 nm using a microplate reader 29. The colony formation assay was performed to measure cell proliferative capacity and sensitivity to trastuzumab. Every 2*10^3 cells were cultured in one well of the 6-well plates for 3 weeks, fixed with polyformaldehyde (POM), and stained with crystal violet. The Cell-Light EdU Apollo *In Vitro* Kit (Ribobio, China) was used to detect cell proliferation.

### Tumor xenograft and drug tolerance models

For each mouse, a total of 2*10^7 tumor cells were subcutaneously into the left groin of 4-week-old BALB/c (nude). Then, the mice were grouped to receive wild-type SKBR3 cells (control), SKBR3 cells overexpressing *tRF-27* (tRF-27 OE), SKBR3 cells expressing blank control (tRF-27 NC), and SKBR3 cells with knocked down *tRF-27* (tRF-27 IN). At day 7 after tumorization, the latter three groups were injected with trastuzumab. Tumor growth was regularly monitored. The tumors were collected at day 28.

### RNA pull-down assay and silver staining

RNA was transcribed *in vitro* using the RNAmax-T7 transcription kit (Ribobio, China) and biotinylated using the Pierce RNA 3' End Desthiobiotinylation Kit (Thermo Fisher Scientific, USA). A Pierce Magnetic RNA-Protein Pull-Down Kit (Thermo Fisher Scientific, USA) was used to perform RNA pull-down assay 30. The protein was extracted from streptavidin magnetic beads. Part of them were sent to Nanjing Medical University for LC/MS analysis. The remainder was subjected to Western blotting and silver staining. The Fast Silver Stain Kit (Beyotime, China) was used for silver staining.

### Western blotting

The cells were harvested and lysed in ice-cold buffer (Beyotime, China). Cell lysates were separated by sodium dodecyl sulfate-polyacrylamide gel electrophoresis (SDS-PAGE), and proteins were transferred to polyvinylidene difluoride (PVDF) membranes (Merck Millipore, USA). Target proteins were detected and quantified using a grayscale ratio with Tanon 4600 Series chemiluminescence/fluorescence image analysis system (Tanon, China). Antibody information is provided in the additional file (Table S1).

### RNA immunoprecipitation (RIP) assay

RIP assay was performed using the Magna Nuclear RIP (Native) Nuclear RNA-Binding Protein Immunoprecipitation Kit (Merck Millipore, USA). Antibody information is provided in the additional file (Table S1).

### Immunofluorescence (IF)

Cells were seeded on glass coverslips and cultured for different times based on the objective of the experiments. Then, the cells were double-stained, and DAPI was used as a nuclear counterstain. Images were obtained using a confocal laser scanning microscope. Antibody information is provided in the additional file (Table S1).

### Dataset analysis

The Cancer Genome Atlas (TCGA) data (TCGA-BRCA dataset) and NCBI GEO (GSE76360 dataset) were used to examine the expression of G3BP1 and G3BP2 31. The TCGA-BRCA dataset was divided into 5 subtypes based on the PAM50 in Genefu R package 32. GSE76360 dataset included the data before and after treatment in 50 patients receiving neoadjuvant therapy. mRNA expression of G3BP1 and G3BP2 was obtained. The raw sequencing reads were processed via the DESeq2 R package.

### Reagents and tagged plasmids

The expression data about G3BPs were obtained from UniPort PDB files were downloaded from Swiss-Model. Eight plasmids were used, including two full-length plasmids and six plasmids to construct truncated bodies. The plasmids were constructed by Corues Biotechnology (China). Sequences included digestion sites, Kozak sequences, CDS of interest, and N-terminal HA tags. The plasmids were transfected into the cells according to the manufacturer's instructions.

### Co-immunoprecipitation (co-IP) assay

Co-immunoprecipitation (co-IP) assay was performed using an Abcam and BeyoMag™ Protein A+G magnetic beads (Beyotime, China). Briefly, the cell lysate was co-incubated with antibody-linked magnetic beads, and the protein was eluted from the magnetic beads. Antibody information is provided in the additional file (Table S1).

### Purification of full-length and truncated HA-G3BP2 proteins

Plasmids were transfected into HEK-293T cells to express full-length and truncated HA-G3BP2 proteins. The protein purification was performed using BeyoMag™ Anti-HA Affinity Gel (Beyotime, China). The Fast Silver Stain Kit (Beyotime, China) was used for silver staining.

### RNA-electrophoretic mobility shift assays (RNA-EMSA)

RNA-EMSA was performed to demonstrate the ability of RNA to bind to proteins *in vitro*. The biotin-labbed *tRF-27* and purified full-length and truncated HA-G3BP2 proteins were used for RNA-EMSA. BeyoMag™ Chemiluminescent RNA EMSA Kit (Beyotime, China) was uesd under the the manufacturer's instructions.

### Predicting molecular interactions

The expression data of G3BPs were obtained from UniPort. PDB files were downloaded from Swiss-Model. HDOCK was used to predict the binding of *tRF-27* to NTF2 domain in G3BPs. The results were demonstrated using PyMOL.

### Immunohistochemistry (IHC)

The clinical samples of patients and tumor samples of the mice were analyzed by immunohistochemistry. In all, 4 μm formalin-fixed and paraffin-embedded sections were de-paraffinized with xylene twice, and rehydrated in a cascade of 100%, 90%, 80%, and 70% alcohol solution. The antigens were retrieved with Tris-EDTA buffer for 3-5 min at 100 °C. The sectios were blocked with 3% hydrogen peroxide solution for 10 min and then with 5% bovine serum albumin (Sigma) for 30 min, incubated with primary antibodies overnight, and analyzed using the ChemMate EnVision kit (Dako, Carpinteria, CA, USA). The investigators were blinded to group allocation during the experiments.

### Statistical analysis

Data from more than three independent experiments were presented as mean ± SD. Student's *t* test was used for pairwise comparisons between groups, and one-way ANOVA for comparisons among three or more groups. The chi-squared test was used for the data of clinical samples. All experiments were performed independently at least thrice. All statistical analyses were performed using GraphPad Prism software or SPSS software. P < 0.05 indicated statistical significance.

## Results

### *tRF-27-ZDXPHO53KSN* predicted trastuzumab resistance in breast cancer

**Figure 1:**
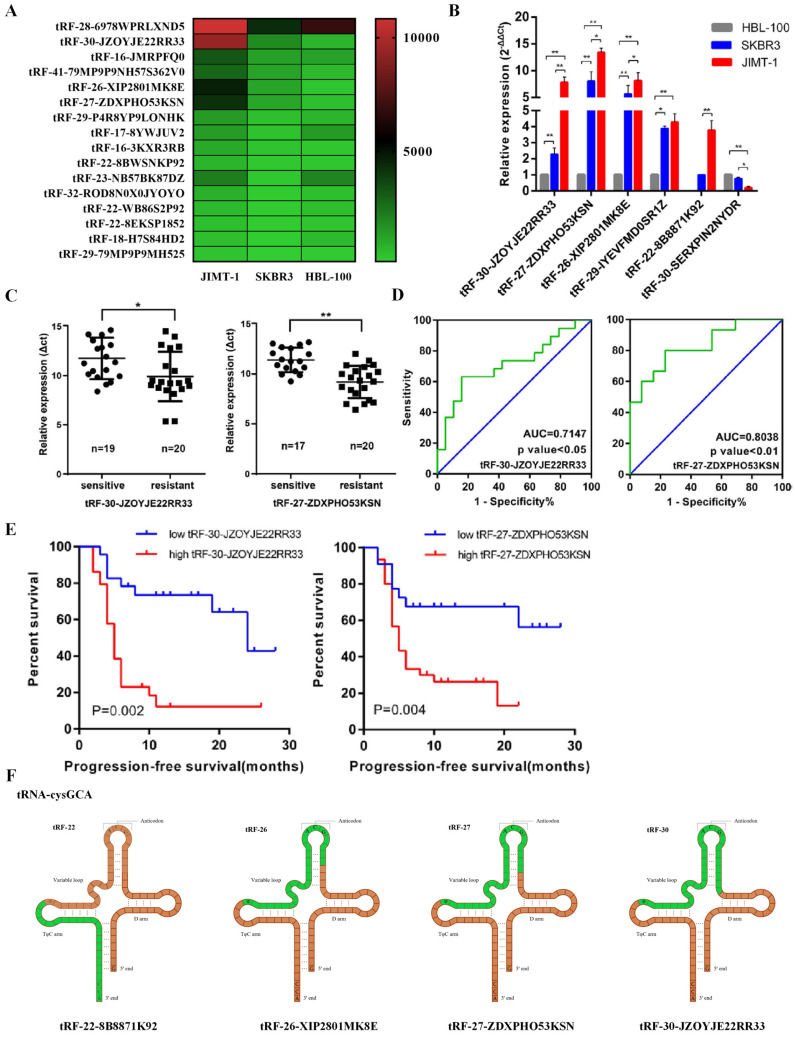
*** tRF-27-ZDXPHO53KSN* predicted trastuzumab resistance of breast cancer. A**. RNA sequencing was used to measure the expression levels of tRNA-derived fragments in cell lines HBL-100, SKBR3, and JIMT1. The top 20 significant tRNA-derived fragments were selected to draw a heatmap. **B**. QRT-PCR analysis of *tRF-30-JZOYJE22RR33*, *tRF-27-ZDXPHO53KSN*, *tRF-26-XIP2801MK8E*, *tRF-29-IYEVFMD0SR1Z*, *tRF-22-8B8871K92*, and *tRF-30-SERXPIN2NYDR* expression in HBL-100, SKBR3 and JIMT-1 cells. Gene expression was normalized to that of *U6* expression, and fold changes by comparing gene expression levels to those in HBL-100 cells were calculated using the 2 ^-△△Ct^ method. **C**. Serum was sampled from 28 trastuzumab-sensitive patients and 29 trastuzumab-resistant patients, total RNA was extracted, and the expression levels of *tRF-30-JZOYJE22RR33* and *tRF-27-ZDXPHO53KSN* were measured with qRT-PCR. **D**. A ROC curve analysis showed the ability of *tRF-30-JZOYJE22RR33* and* tRF-27-ZDXPHO53KSN* to predict trastuzumab resistance. **E**. Progression-free survivals (PFSs) in the 52 metastatic HER-2 positive breast cancer patients who received advanced trastuzumab treatment. Patients with high expression *of tRF-30-JZOYJE22RR33* and *tRF-27-ZDXPHO53KSN* showed worse PFSs. **F**. *tRF-30-JZOYJE22RR33*, *tRF-22-8B8871K92*, *tRF-26-XIP2801MK8E* and *tRF-27-ZDXPHO53KSN* originate from the same digestion site of *tRNA-cysgca*.

In previous studies, we have focused on the role of tRNA-derived fragments in trastuzumab resistance in breast cancer 21. We selected three cell lines, including trastuzumab-sensitive cell line SKBR3, the trastuzumab-resistant cell line JIMT1, and HBL-100 from breast epithelial cells as controls, for RNA sequencing (Figure 1A). In our study, 36 upregulated and 21 downregulated tRNA-derived fragments were screened between HBL-100 and SKBR3/JIMT1 cell lines, as well as 11 upregulated and one downregulated between SKBR3 and JIMT1 cell lines. Based on these results, we selected five upregulated genes (*tRF-30-JZOYJE22RR33*, *tRF-27-ZDXPHO53KSN*, *tRF-26-XIP2801MK8E*, *tRF-29-IYEVFMD0SR1Z*, and *tRF-22-8B8871K92*) and one downregulated gene (*tRF-30-SERXPIN2NYDR*) for subsequent studies.

qRT-PCR confirmed that the expression levels of the tRNA-derived fragments were consistent with the sequencing results (Figure 1B). We then collected serum from 28 trastuzumab-sensitive and 29 drug-resistant patients, extracted total RNA, and measured the expression levels of these tRNA-derived fragments using qRT-PCR. Combined with their clinical data, we identified that *tRF-30-JZOYJE22RR33* and *tRF-27-ZDXPHO53KSN* were sensitive to predict trastuzumab resistance (Figure 1C and 1D). Progression-free survival (PFS) analysis also showed that patients with high expression of these two tRNA-derived fragments had a worse PFS (Figure 1E).

We then analyzed the sources of these tRNA-derived fragments. According to the data analysis of MINTbase, we found that both *tRF-30-JZOYJE22RR33* and *tRF-27-ZDXPHO53KSN* originated from the same digestion site of* tRNA-cysgca*, with only difference in three bases (Figure 1F) 26. At the same time, *tRF-27-ZDXPHO53KSN* had a stronger ability to predict drug resistance than *tRF-30-JZOYJE22RR33*; therefore, we chose *tRF-27-ZDXPHO53KSN* (hereinafter referred to as* tRF-27*) as our research object.

### *tRF-27* overexpression enhanced the resistance of HER2 positive breast cancer cells against trastuzumab

**Figure 2:**
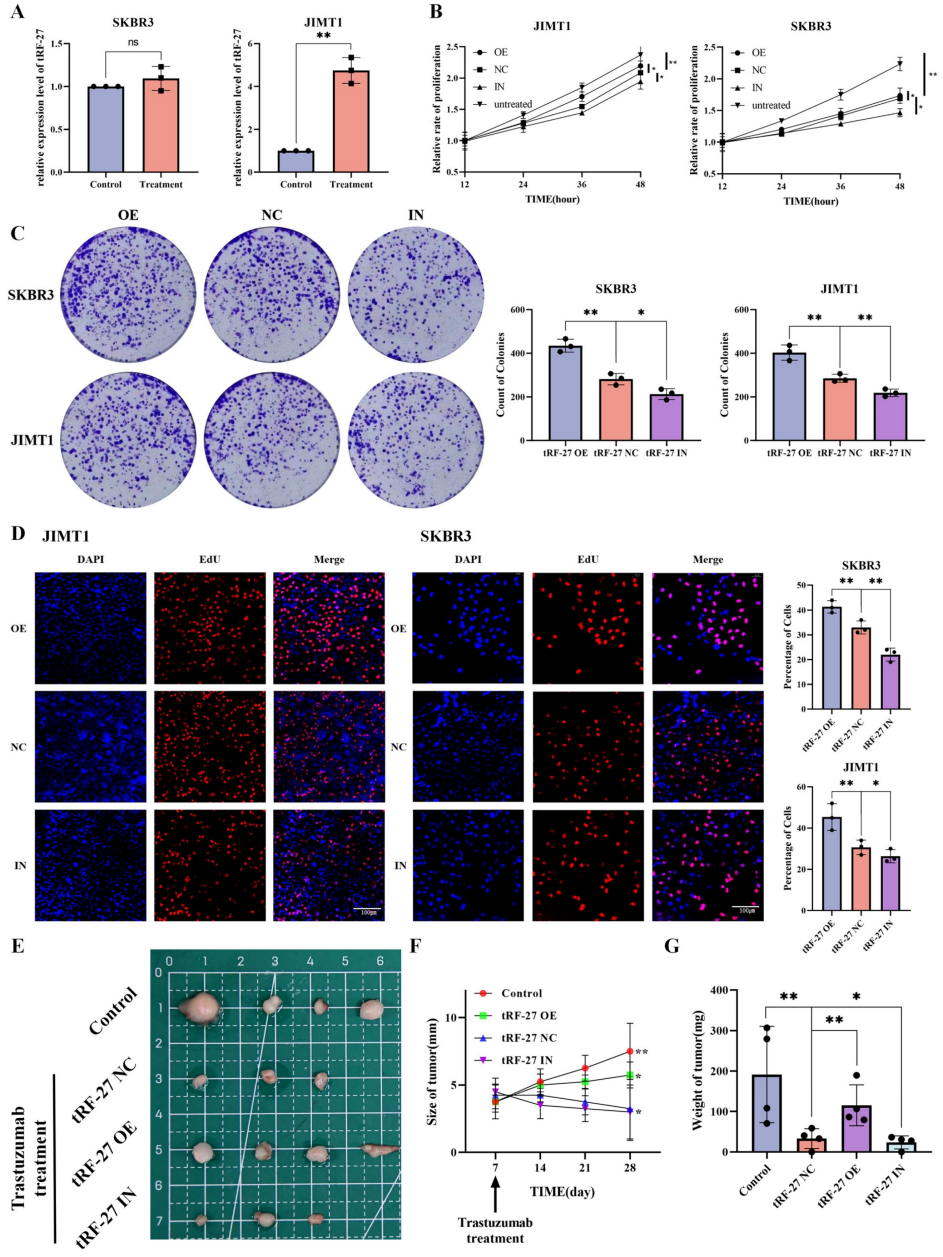
*** tRF-27* affected trastuzumab resistance of HER2 positive breast cancer cells. A**. The expression levels of *tRF-27* in trastuzumab-sensitive SKBR3 cells and trastuzumab-resistant JIMT1 cells under trastuzumab stimulation by qRT-PCR. Data were expressed as mean ± SEM; *P<0.05, **P<0.01, ***P<0.001. **B**. CCK-8 was performed to detect cell growth and survival. Under the stimulation of trastuzumab, the growth rate of SKBR3 cells decreased significantly, but less significantly in JIMT1 cells. Stimulated by trastuzumab, the growth rate of cells overexpressing* tRF-27* increased, and knocking down *tRF-27* inhibited cell growth. **C**. Cell colony formation assay was performed. The colony-forming ability of cells was positively correlated with the expression level of* tRF-27*. **D**. EdU assay was used to illustrate the proliferative state of cells. Overexpression of *tRF-27* in both JIMT1 and SKBR3 cell lines significantly increased the proliferation, while knocking down *tRF-27* inhibited this proliferation. **E**. Tumor xenograft in nude mice. 2*10^7 tumor cells were injected subcutaneously into every left groin of BALB-c(nude) mice which were divided into 4 groups (n=4 in each group), including groups receiving wild-type SKBR3 cells (control), SKBR3 cells overexpressing *tRF-27* (tRF-27 OE), SKBR3 cells expressing blank control (tRF-27 NC), and SKBR3 cells knocking down *tRF-27* (tRF-27 IN). At day 7 of subcutaneous tumorization, the latter 3 groups were injected with trastuzumab. Tumors were collected at day 28 and one tumor disappeared in one mouse of the tRF-27 NC group, and one tumor disappeared in one mouse of the tRF-27 IN group. **F - G**. Regularly monitoring of tumor size (mm), and tumor weigh (mg). Trastuzumab greatly inhibited tumor growth. High expression levels of* tRF-27* enhanced tumor resistance.

To further investigate the significance of *tRF-27* in breast cancer drug resistance, we first compared its expression levels in trastuzumab-sensitive SKBR3 and trastuzumab-resistant JIMT1 cells by qRT-PCR (Figure 2A). As suggested in previous studies, the concentration of trastuzumab added to the medium in our study was 2 μg/ml, which is similar to the clinical concentration 4. We found that* tRF-27* expression was significantly upregulated in JIMT1 cells stimulated with trastuzumab; in contrast, this level was almost unchanged in SKBR3 cells. We transfected JIMT1 and SKBR3 cells with the designed lentiviruses to construct *tRF-27* overexpression and knockdown cell lines for subsequent studies. Trastuzumab mainly inhibits the growth of tumor cells by reshaping the immune environment 33. We used the level of cell growth and proliferation in a trastuzumab-stimulating environment to reflect the level of tolerance of cells to trastuzumab 34. The CCK-8 assay was used to detect cell growth and survival. Upon stimulation with trastuzumab, the growth rate of SKBR3 cells decreased significantly. The growth of JIMT1 cells was not changed evidently. This is consistent with our previous findings. Stimulated by the trastuzumab, we compared the growth rates of cells with overexpressed or knocked down *tRF-27*. *tRF-27* overexpression increased, but *tRF-27* knockdown decreased the growth rate of cells (Figure 2B). Cell colony formation assay showed that the colony formation rate of cells was positively correlated with the expression level of *tRF-27* (Figure 2C). EdU assay showed that *tRF-27* overexpression significantly increased, but *tRF-27* knockdown inhibited the proliferation in both JIMT1 and SKBR3 cells (Figure 2D). These results suggested that overexpression of *tRF-27* could promote the growth of cells stimulated by trastuzumab, thus enhancing the tolerance of cells to trastuzumab.

We validated our hypothesis using tumor xenografts in nude mice. We injected 2*10^7 tumor cells subcutaneously into the left groin of BALB/c (nude) of each mouse. The mice were divided into four groups (four in each group), respectively treated with wild-type SKBR3 cells (control), SKBR3 cells overexpressing *tRF-27* (tRF-27 OE), SKBR3 cells expressing blank control (tRF-27 NC), and SKBR3 cells with knocked down* tRF-27* (tRF-27 IN). At day 7 after subcutaneous tumourisation, the latter three groups of mice were injected with trastuzumab. At day 28, the tumor disappeared in the tRF-27 NC group and the tRF-27 IN group (Figure 2E). The growth of tumor in the remaining groups proven the role of *tRF-27* in promoting drug resistance (Figure 2F and 2G). Taken together, overexpression of *tRF-27* could enhance trastuzumab resistance in breast cancer tumor cells.

### *tRF-27* bound to G3BPs and acted independent of stress granules

**Figure 3:**
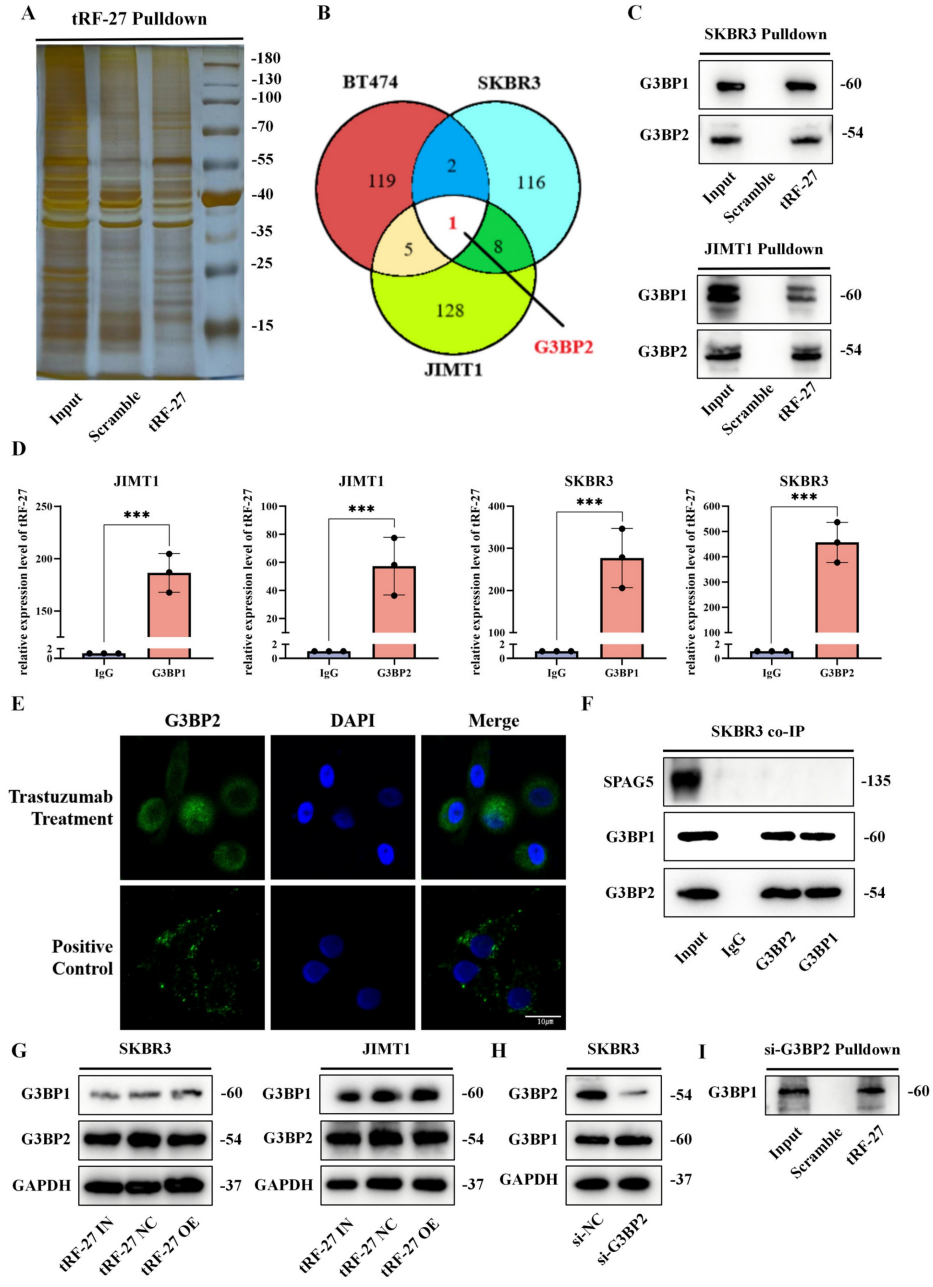
***tRF-27* bound to G3BPs and functioned independent of stress granules. A**. SKBR3, JIMT1 and BT474 cells were used for tRF-27 RNA pull-down assay. Silver-stained bands of JIMT1 cells. **B**. Intersection between SKBR3, JIMT1 and BT474 after respectively removing the intersections in the *tRF-27* group (tRF-27) and the scramble RNA group (Scramble) in the mass spectrometry results. Only one protein G3BP2 was bound simultaneously in all three cell lines. **C.** We tested G3BPs expression with Western blotting. In SKBR3 and JIMT1 cells, both G3BP1 and G3BP2 could bind to *tRF-27*. **D**. RNA immunoprecipitation (RIP) assay with antibodies of G3BP1 and G3BP2. t*RF-27* bound to G3BP1/G3BP2 in JIMT1 and SKBR3 cells confirmed by qRT-PCR. **E.** Immunofluorescence assay of G3BP2 in SKBR3 cells; stress granules formed in a nutrient-deficient environment (Positive control), while the trastuzumab group did not show nucleated stress granules (Trastuzumab treatment). **F**. Co-immunoprecipitation (co-IP) with G3BPs; SPAG5 did not bind to G3BPs. **G**. Overexpressing (tRF-27 OE) and knocking down (tRF-27 IN) *tRF-27* did not alter the expression level of G3BPs. **H**. Knockdown of G3BP2 and the expression levels of G3BP1 in cells. **I**. Another RNA pull-down assay was performed with G3BP2 knockdown cells; *tRF-27* still bound to G3BP1.

To determine the mechanism by which *tRF-27* exerts its biological functions, we constructed biotin-labeled *tRF-27* probes and scramble probes for pull-down assay 35. SKBR3, JIMT1 and drug-sensitive BT474 cell lines were used for the experiment. Having been cultured with trastuzumab for 48 h, 2*10^8 cells of each type of cells were collected for pull-down assay (Figure 3A and S1A). The obtained protein was silver-stained and detected using mass spectrometry 36. The mass spectrometry results are appended to the Supplementary Materials (Table S2). The intersection in pull-down assays results of the *tRF-27* group and the scramble RNA group was removed (Figure 3B and S1B). We found that only one protein, G3BP2, was bound simultaneously in all three cell lines 15. We found that G3BP2 and G2BP1 share 70% homologous sequences (Figure S1C) 19; therefore, we tested their expression with Western blotting. In SKBR3 and JIMT1 cell lines, both G3BP1 and G3BP2 could bind to *tRF-27* (Figure 3C). In SKBR3 and JIMT1 cell lines, RNA immunoprecipitation (RIP) assay was performed with antibodies against G3BP1 and G3BP2. The RNA obtained was detected by qRT-PCR. The results confirmed the binding pattern (Figure 3D).

G3BPs serve as key proteins in stress granules, and function in various environments 17, 20. When stress granules form, G3BPs can competitively bind to regulatory associated protein of MTOR complex 1 (RPTOR), a key protein of the MTORC1 complex through sperm associated antigen 5 (SPAG5, also known as Astrin) 37, thereby inhibiting the activation of MTORC1 by riveting the TSC complex on the surface of lysosomes without stress granules. Abnormal activation of the PI3K/AKT/mTOR pathway is engaged in trastuzumab resistance 38. This information moved us to explore an association between G3BPs and trastuzumab resistance.

First, we clarified whether trastuzumab causes the formation of stress granules 39. We cultured SKBR3 and JIMT1 cells in a trastuzumab-exposed environment and a glucose-deficient environment, considering that nutrient deficiency was a clear trigger for the formation of stress granules 40. In immunofluorescence assay, stress granules formed in a nutrient-deficient environment, whereas the trastuzumab-treated group did not show nucleated stress granules (Figure 3E and S2A). We collected SKBR3 cells stimulated by trastuzumab for co-IP assay, and found that SPAG5 did not bind to G3BPs (Figure 3F). Western blotting was used to detect the expression levels of G3BPs in cells with* tRF-27* overexpression or knockdown. Similarly, *tRF-27* did not alter the expression levels of G3BPs in cells stimulated by trastuzumab (Figure 3G) 19. This indicated that stress granules did not form under the stimulation of trastuzumab. In addition, *tRF-27* might function in a manner independent of stress granules.

We constructed a cell line for G3BP2 knockdown using siRNAs, and found that G3BP2 knockdown did not significantly increase G3BP1 expression (Figure 3H). The cells with G3BP2 knockdown were subjected to another RNA pull-down assay, and we found that* tRF-27* still bound to G2BP1(Figure 3I). This suggested that *tRF-27* could bind directly to the two proteins without relying on the dimeric relationship of G3BPs.

### G3BPs were stably expressed in breast cancer cells, but G3BPs overexpression reduced trastuzumab tolerance

Although the regulatory mechanism of G3BPs in the MTORC1 signaling pathway has been well understood, evidence still lacks that G3BPs are involved in trastuzumab resistance. It has proven that the MTORC1 signaling pathway is repressed as the expression of G3BPs increases, thus inhibiting the growth and proliferation of cells 20. The abnormal activation of the PI3K/AKT/mTOR signaling pathway is essential for trastuzumab resistance. Here, we obtained breast cancer-related data from the TCGA database and used the Genefu R package to classify all patients into 5 subtypes according to PAM50. Interestingly, we found that the expression levels of G3BP1 and G3BP2 did not vary across the five subtypes (Figure S2B). In the GSE76360 of the GEO database, by comparing the pre- and post-treatment sequencing data of 50 patients who received neoadjuvant trastuzumab-containing regimens, we found no significant changes in G3BP1 and G3BP2 expression before and after treatment (Figure S2C).

We constructed siRNA of G3BP2 and transfected it into SKBR3 cells. A plasmid overexpressing G3BP2 was constructed and transfected into SKBR3 cells. All experimental and control cells were cultured under trastuzumab exposure. Cell growth was detected by colony formation and EdU assays (Figure S3A and S3B). We found that G3BP2 knockdown promoted, but G3BP2 overexpression inhibited the growth and proliferation of cells.

Western blotting was used to detect protein expression in cells exposed to trastuzumab (Figure S3C). We found that G3BP2 knockdown promoted phosphorylation of Tuberin, but inhibited the phosphorylation of substrate eukaryotic translation initiation factor 4E binding protein 1 (4EBP1) of MTORC1. G3BP2 overexpression inhibited phosphorylation of Tuberin and promoted phosphorylation of 4EBP1^19^. Taken together, G3BP2 could inhibit cell growth under trastuzumab exposure, suggesting that* tRF-27* might affect the function but not the expression of G3BPs.

### *tRF-27* bound to G3BPs through a specific structure

**Figure 4:**
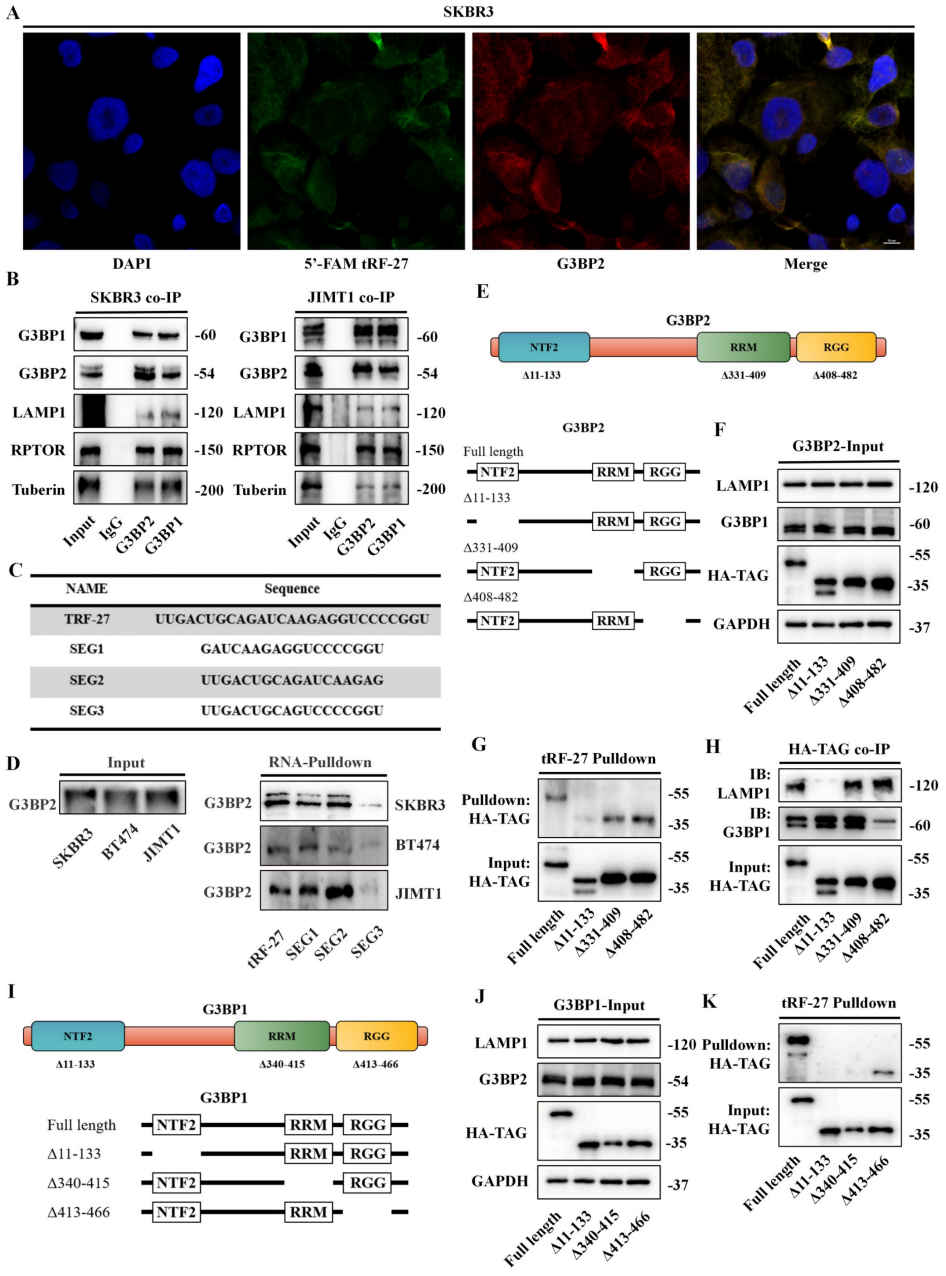
*** tRF-27* bound to G3BPs through a specific structure. A**. We transfected *tRF-27* with 5'-FAM into SKBR3 cells, and G3BP2 protein was immunofluorescence-stained; both were predominantly located in the cytoplasm. **B**. Co-IP with G3BP1/G3BP2 was performed; G3BPs combined with LAMP1, RPTOR and Tuberin. **C**. The sequences of the probes designed for RNA pull-down assay. **D**. Trastuzumab-stimulated cells were collected for pull-down assays with designed probes. Loss of the middle 9 bases damaged the ability of the probes to bind to G3BP2. **E.** The protein structure of G3BP2. We constructed plasmids with HA-TAG tags expressing G3BP2 that lost these three domains. **F**.Full-length and truncated G3BP2 do not affect the expression levels of G3BP1 and LAMP1. **G.** Plasmids of full-length and truncated G3BP2 were transfected into HEK293T cells. *tRF-27* pull-down assay was performed.* tRF-27* did not bind to G3BP2 protein that lost the NTF2 domain. **H**. Co-IP showed that LAMP1 was also integrated into NTF2 domain. The binding between G3BP1 and G3BP2 was more likely to depend on the RGG domain. **I.** The protein structure of G3BP1. We constructed plasmids with HA-TAG tags expressing G3BP1 that lost these three domains. **J**. Full-length and truncated G3BP1 do not affect the expression levels of G3BP2 and LAMP1. **K**. *tRF-27* did not bind to the G3BP2 protein that is missing the NTF2 or RRM domain.

Because stress granules were not formed, we suspected that G3BPs function through another pathway. G3BPs, as connexins, rivet lysosomal membrane protein LAMPs and TSC complex key protein Tuberin (also known as TSC2), thereby activating the TSC complex and inhibiting MTORC1. We transfected *tRF-27* with 5'-FAM into SKBR3 and JIMT1 cells and immunofluorescently stained G3BP2 protein, finding that both *tRF-27* and G3BP2 were predominantly located in the cytoplasm (Figure 4A and S4A). First, we verified the bindings between these proteins. Proteins were extracted from cells cultured in a trastuzumab-stimulated environment and co-immunoprecipitated. G3BPs combined with LAMP1, RPTOR and Tuberin (Figure 4B). This finding is consistent with the results of previous studies, indicating that G3BPs function through this pathway when stimulated by trastuzumab.

We further explored the binding regions between *tRF-27* and G3BPs. According to the sequence of *tRF-27*, we designed three fragments through missing the front, back, and middle nine bases. Biotin-labeled probes were constructed (Figure 4C). Drug-stimulated cells were collected for Pull-down assay (Figure S4B). The results showed that the ability of the probe to bind to G3BP2 was greatly reduced when the middle nine bases were missed, suggesting that *tRF-27* relies on the middle nine bases to bind to G3BP2 (Figure 4D). Online docking tools were used to predict the spatial structure of these four RNAs (Figure S4C) 41. It was found that the complete* tRF-27* had a loop, and this configuration was destroyed when* tRF-27* lost its middle nine bases.

The protein structure of G3BP2 was obtained from UniPort (https://www.uniprot.org). We focused on 3 domains in G3BPs. Previous studies confirmed that G3BPs bind to LAMPs in the NTF2 domain. The RGG and RRM domains are considered as the main domains binding to RNAs (Figure 4E). We found that only the NTF2 domain, as the spatial structure of G3BPs, was resolved, and Alphafold did not provide predictions (Figure S4D) 42, 43. TMHMM was used to predict the transmembrane regions of LAMP1 (Fig S4E). We also predicted the combined model of LAMP1 and G3BP2 (Fig S4F). Based on this, we constructed plasmids with HA-TAG tags expressing G3BP2 and lacking these three domains 44. Full-length and truncated G3BP2 plasmids were transfected into HEK293T cells, and Pull-down and co-IP assays were performed (Figure S5A and S5B). Full-length and truncated G3BP2 do not affect the expression levels of G3BP1 and LAMP1 (Figure 4F). We found that *tRF-27* did not bind to the G3BP2 which lacked the NTF2 region (Figure 4G). Consistent with previous reports, LAMP1 was integrated into this domain (Figure 4H). Binding between G3BP1 and G3BP2 was more likely to depend on the RGG domain. To confirm our results, we constructed truncated G3BP1 bodies (Figure 4I and S5C). Full-length and truncated G3BP2 plasmids were transfected into HEK293T cells, parallelly, full-length and truncated G3BP1 do not affect the expression levels of G3BP2 and LAMP1 (Figure 4F). We found that *tRF-27* could not bind to G3BP1, regardless of the absence of NTF2 or RRM (Figure 4K).

**Figure 5:**
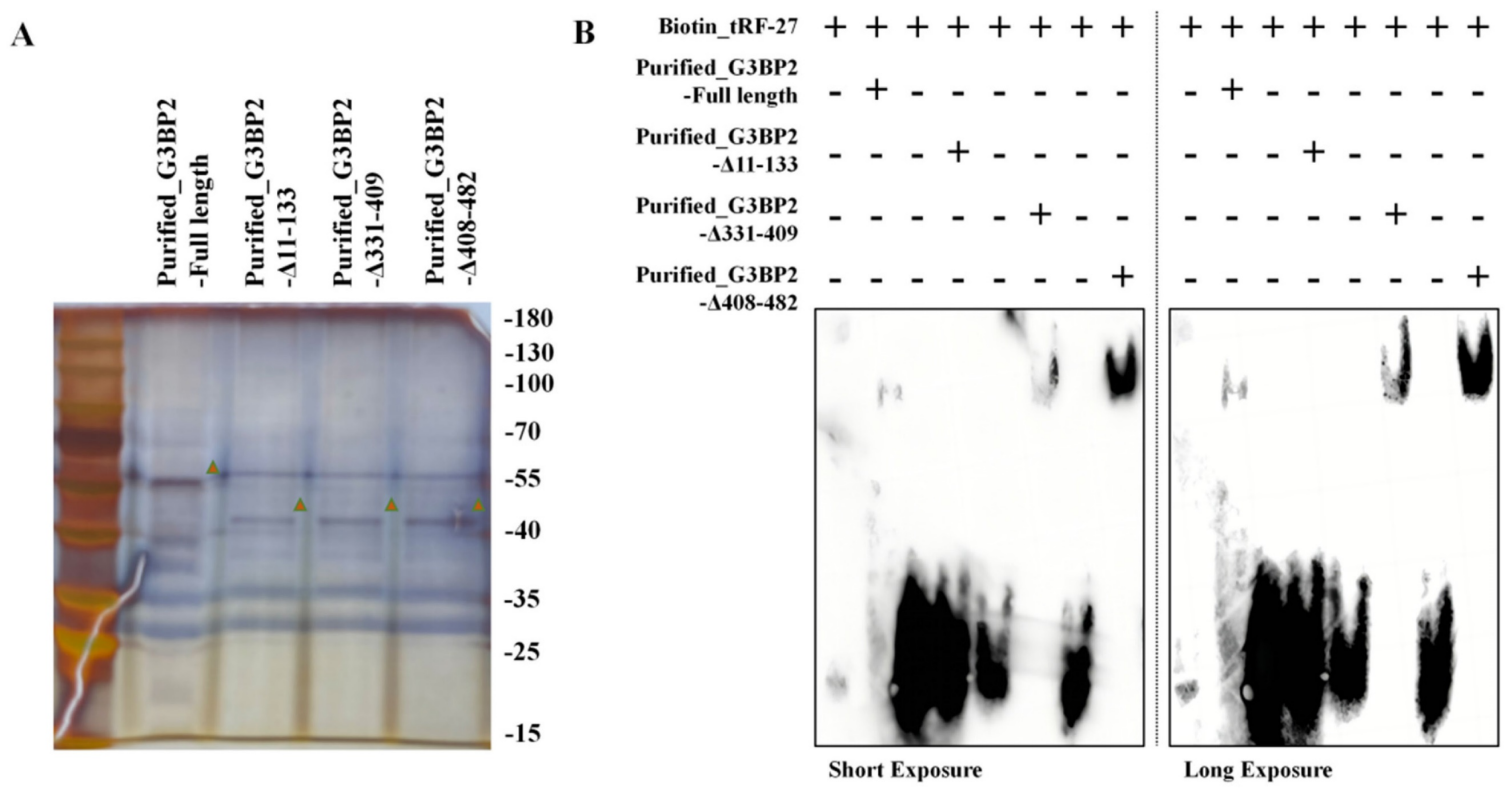
***tRF-27* binds to G3BP2 protein *in vitro.*** Purified full-length and truncated HA-G3BP2 proteins. **B**. RNA-EMSA was performed to demonstrate the ability of RNA to bind to proteins *in vitro*. *tRF-27* binds to the purified G3BP2 and other truncated recombinant proteins, but not to protein that lack the NTF2 domain.

RNA-EMSA was performed to demonstrate the ability of RNA to bind to proteins *in vitro*. We transfected plasmids into HEK-293T cells to express full-length and truncated HA-G3BP2 proteins. The protein purification was performed using Anti-HA Affinity Gel (Figure 5A). The biotin-labbed *tRF-27* and purified full-length and truncated HA-G3BP2 proteins were used for RNA-EMSA. Though observing the swift of *tRF-27* on the gel, we found that *tRF-27* binds to the purified G3BP2 and other truncated recombinant proteins, but not to protein that lack the NTF2 domain (Figure 5B). This is consistent with the phenomenon we observed in experiments *in vivo*.

**Figure 6:**
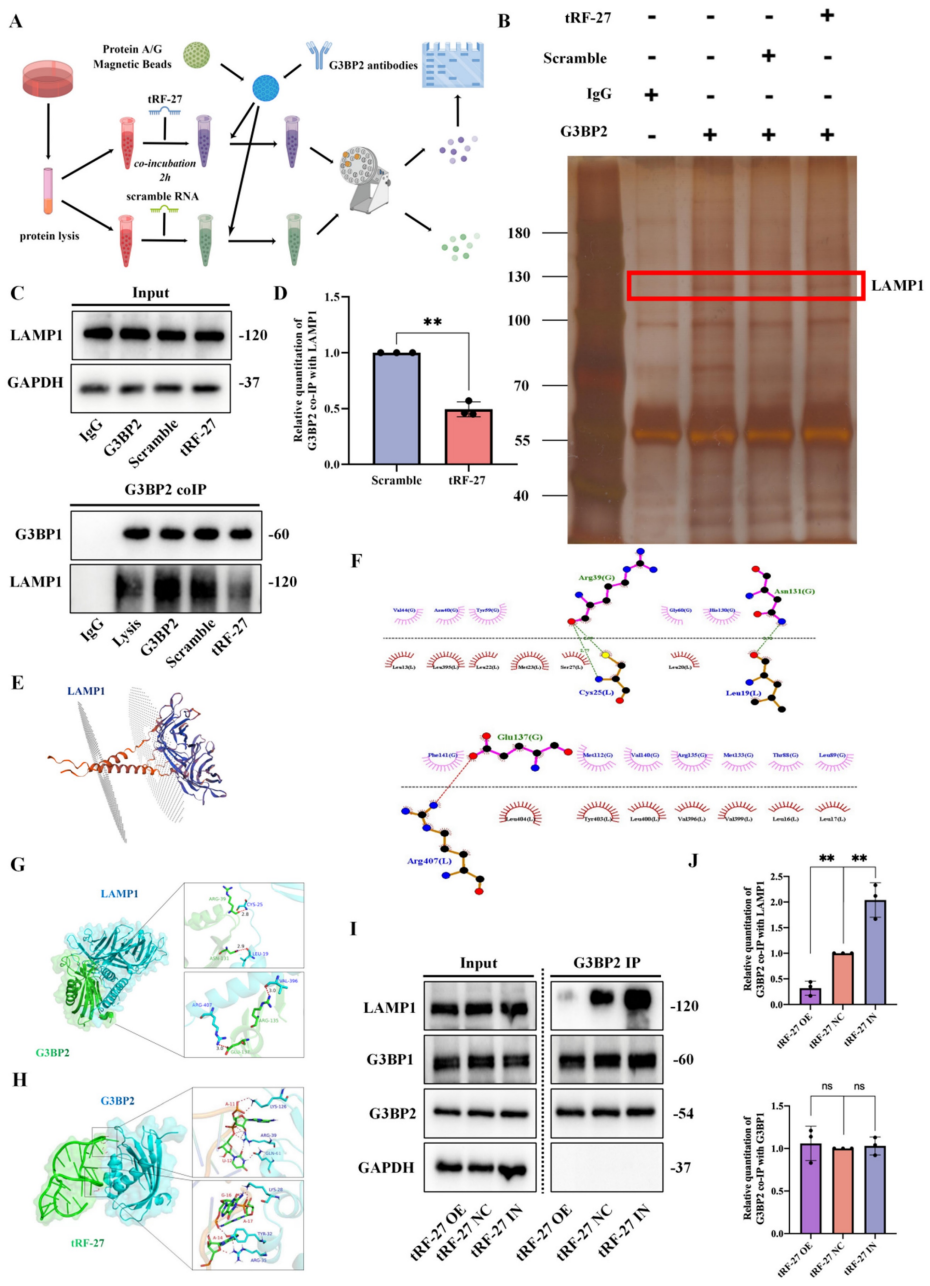
***tRF-27* affected the binding of G3BPs with LAMPs. A**. We synthesized *tRF-27* and fragments of scramble RNA, and incubated two excess RNAs (10 nmol) with equal HEK-293 cell lysate (200 μl of cell lysate, 2*10^7 cells) for 2 h, respectively. Then, the G3BP2 antibody attached to protein A/G magnetic beads were used to perform co-IP, and the protein that could bind to G3BP2 was detected by Western blotting. **B-C**. Silver-stained bands in co-IP. G3BP2 binding to LAMP1 was significantly reduced in lysates co-incubated with *tRF-27*, compared to that in the scramble RNA group. **D.** We repeated the experiment three times, measured and compared the band gray values, and determined that the *tRF-27* group had a significant decrease in the expression of LAMP1, compared to the scramble RNA group. Data were shown as mean ± SEM; *P<0.05, **P<0.01, ***P<0.001. **E**. Transmembrane structure of LAMP1. **F-H**.We used HDOCK to predict the binding of LAMP1 and *tRF-27* to the NTF2 domain in G3BPs, and the results were demonstrated with PyMol. **I-J**. The expression of *tRF-27* did not affect the expression levels of G3BPs and LAMP1. However, when the expression of *tRF-27* increased, the binding of G3BP2 with LAMP1 significantly decreased. The experiment was repeated three times, the band gray values were measured and compared.

This led us to speculate that *tRF-27* might occupy the binding domain of G2BPs and LAMPs, thus affecting the binding of the two. To test this hypothesis, we synthesized *tRF-27* and fragments of scrambled RNA and incubated two excess RNAs (10 nmol) with equal amounts of HEK-293 cell lysate (200 μl of cell lysate, 2*10^7 cells) for 2 h. The G3BP2 antibody-attached protein A/G magnetic beads were used to perform co-immunoprecipitation, and the protein that could bind to G3BP2 was detected by Western blotting (Figure 6A). We found that G3BP2 binding to LAMP1 was significantly reduced in lysates co-incubated with* tRF-27*, compared to that in the scramble RNA group (Figure 6B and 6C). At the same time, as a control, the binding of G3BP2 to G3BP1 did not change significantly. We repeated the experiment three times, measured and compared the band gray values, measured and compared the band quantitation values (Figure 6D). The results showed that the *tRF-27* group had a statistically significant decrease in quantitation, compared with the scramble group. We believed that *tRF-27* might inhibit the ability of G3BPs to connect tubulin and lysosomes by occupying the binding site of G3BPs to LAMPs, thereby relieving the inhibition of TSC complexes on MTORC1 45. We used HDOCK to predict the binding regions of G3BP2 with the cytoplasmic structure of LAMP1 and demonstrated them with PyMOL (Figure 6E-6G). We also predict the binding regions of G3BP2 with *tRF-27* and demonstrated them with PyMOL (Figure 6H). We confirmed that G3BP2 combinds with LAMP1 and *tRF-27* at the same binding site. SKBR3 cells overexpressing and knocking down *tRF-27* were collected for another immunoprecipitation. The expression of *tRF-27* did not affect the expression levels of G3BPs and LAMP1. However, when the expression of *tRF-27* increased, the binding of G3BP2 with LAMP1 significantly decreased (Figure 6I). We repeated the experiment three times, measured and compared the band gray values, measured and compared the band quantitation values (Figure 6J).

Functional rescue experiments were also conducted. G3BP2-overexpressing plasmids were transfected into SKBR3 cells in a trastuzumab-exposed environment. Cell growth was detected by colony formation essay (Figure S5D). EdU assay showed that the proliferation of cells in the overexpression* tRF-27* group was significantly inhibited, but not in the overexpression of G3BP2 which lacked NTF2 (Figure S5E).

### *tRF-27* promoted the activation of MTORC1

Consistent with previous reports, we found that trastuzumab inhibited the entire PI3K/AKT/mTOR signaling pathway in SKBR3 cells. Abnormal activation of this signaling pathway was observed in drug-resistant cells (Figure S6A and S6B).

**Figure 7:**
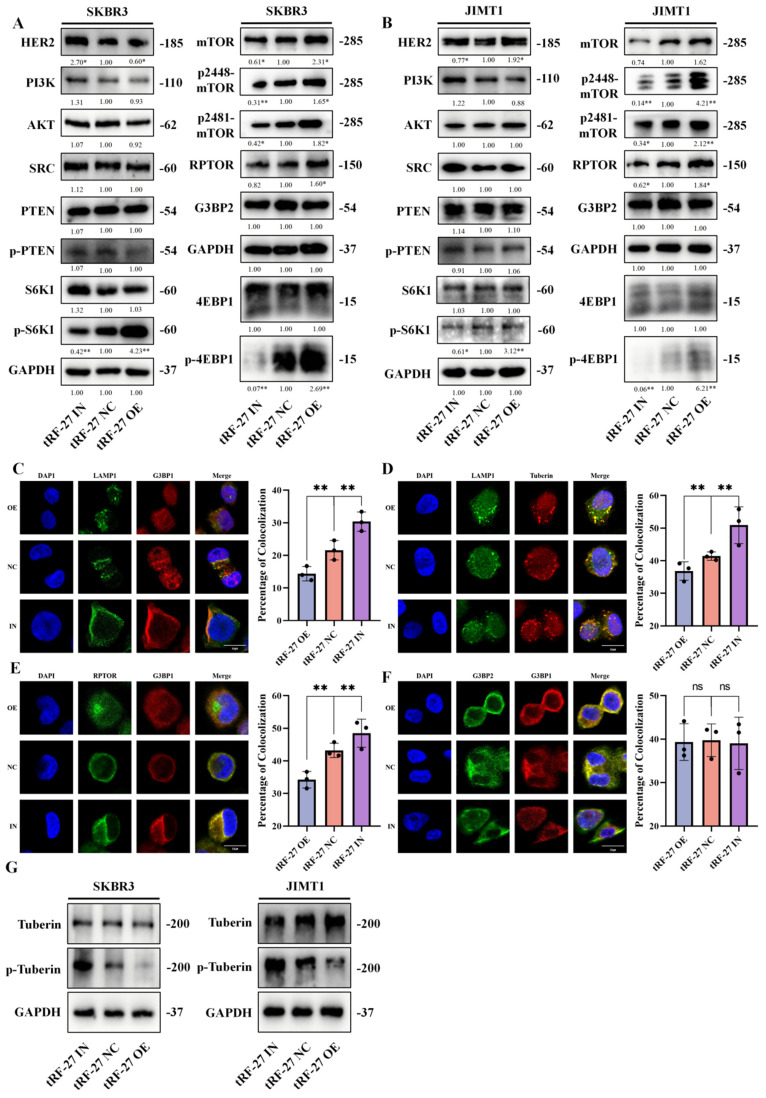
***tRF-27* promoted the activation of MTORC1. A-B.** The expression levels of PI3K/AKT/mTOR signaling pathway proteins were detected in cells with *tRF-27* overexpression (tRF-27 OE) and knockdown *tRF-27* (tRF-27 IN) in a trastuzumab-stimulating environment. TRF-27 did not affect the proteins upstream MTORC1, but significantly promoted MTORC1 activation. **C-F.** Immunofluorescence with G3BP1,G3BP2, LAMP1, Tuberin and RPTOR. When *tRF-27* was overexpressed, the colocalization of G3BP1 and Tuberin with LAMP1 was hindered. Knocking down *tRF-27* increased the colocalization of G3BP1 and LAMP1. Overexpressing *tRF-27* also separated G3BP1 from RPTOR. **G**. Overexpression of* tRF-27* promoted phosphorylation of Tuberin.

We measured the expression of proteins involved the PI3K/AKT/mTOR signaling pathway in cells with *tRF-27* overexpression and knockdown in a trastuzumab-stimulated environment (Figure 7A and 7B) 46. We found that *tRF-27* did not affect the expression of proteins upstream MTORC1, but significantly promoted MTORC1 activation. This result was consistent with our previous assumptions.

We also treated cells with rapamycin, an inhibitor of MTORC1, in combination with trastuzumab, and found that the effect of *tRF-27* in promoting cell proliferation was largely eliminated (Figure S6C). This result was consistent with our hypothesis.

We further used immunofluorescence to detect the colocalization of proteins. In SKBR3 cells, after* tRF-27* was overexpressed, the colocalization of G3BP1 or Tubulin with LAMP1 was reduced (Figure 7C and 7D). Knockdown of *tRF-27* increased the colocalization of G3BP1 with LAMP1 and increased the colocalization of Tuberin with LAMP1. Overexpression of *tRF-27* spatially separated G3BP1 and RPTOR (Figure 7E), and *tRF-27* could not change the colocolization of G3BP1 and G3BP2 (Fig 7F). Overexpression of *tRF-27* inhibited Tuberin phosphorylation (Figure 7G). This indicated that the TSC complex was activated by *tRF-27* overexpression.

**Figure 8:**
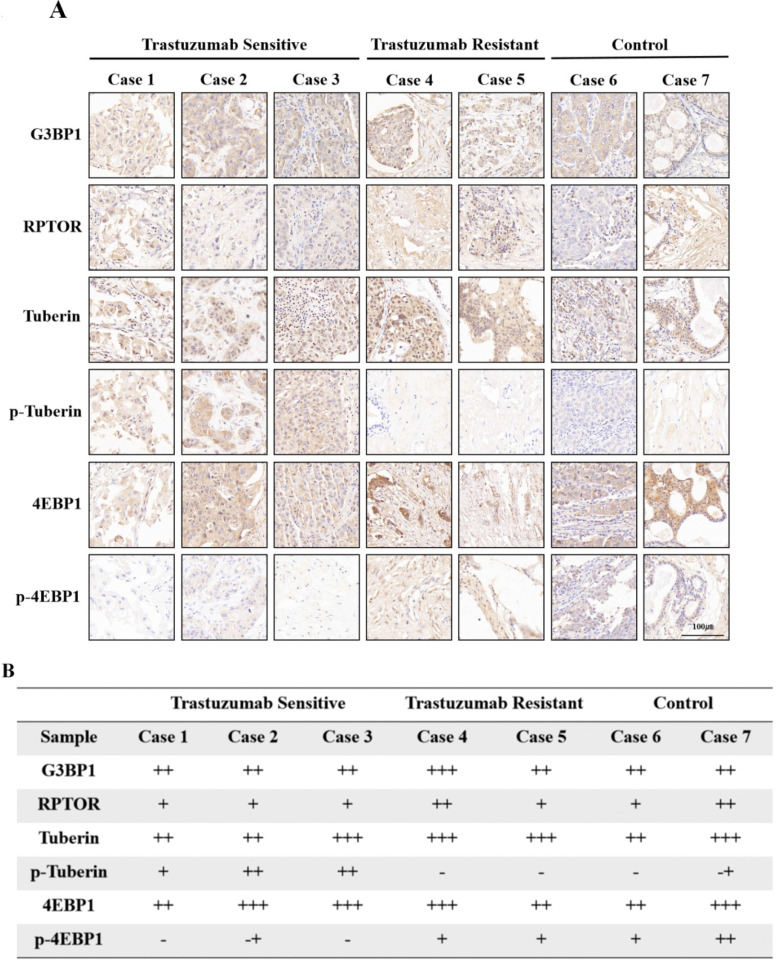
** Phosphorylation of Tuberin was associated with trastuzumab resistance.** Tumor specimens from HER2-positive breast cancer patients who had received trastuzumab-containing regimens were divided into trastuzumab-sensitive and trastuzumab-resistant groups, based on whether the patients' Miller-Payne scores were greater than 2. **A-B**. Tissue samples from two additional patients who had not received neoadjuvant therapy were used as controls. Trastuzumab treatment did not affect G3BP1 expression, but trastuzumab-sensitive patients showed stronger expression of p-Tuberin and relatively weaker expression of p-4EBP1.

To test our hypothesis, we collected tumor samples from HER2-positive breast cancer patients who had received neoadjuvant trastuzumab-containing regimens, and divided the patients into trastuzumab-sensitive and trastuzumab-resistant groups based on whether their Miller-Payne scores were greater than 2. Tissue samples from two additional patients who did not receive neoadjuvant therapy were used as controls. Immunohistochemical analysis showed that trastuzumab treatment did not affect G3BP1 expression, but trastuzumab-sensitive patients showed stronger expression of p-tuberin and weaker expression of p-4EBP1 (Figure 8A and 8B).

**Figure 9:**
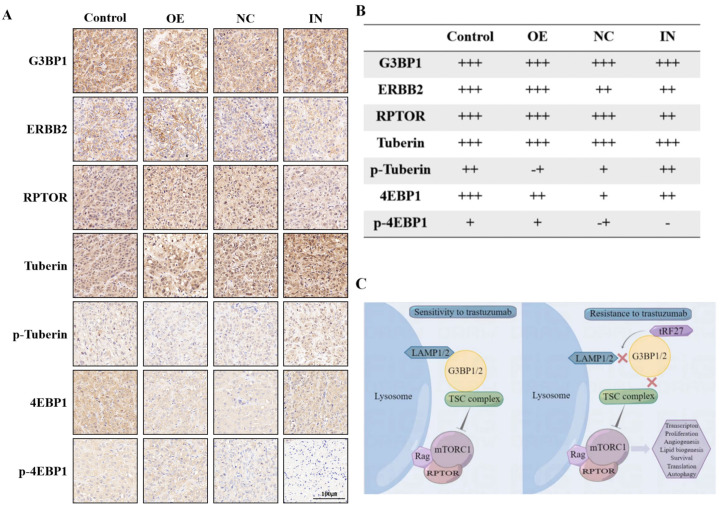
*** tRF-27* promoted the phosphorylation of Tuberin and the activation of MTORC1. A**. Immunohistochemistry of mouse xenograft tumor specimens. **B.** As shown by the immunohistochemical results, overexpression of *tRF-27*(OE) inhibited the phosphorylation of Tuberin and promoted the activation of MTORC1. Inhibition of *tRF-27*(IN) promoted the phosphorylation of Tuberin and inhibited the activation of MTORC1. **C**. High levels of *tRF-27* blocked lysosomal localization of TSC complexes by competitively combining G3BPs with LAMPs. As the inhibition on MTORC1 was relieved, the growth of trastuzumab-stimulated cells was promoted, thus enhancing the tolerance of tumor cells to trastuzumab.

In addition, we detected the expression of these proteins in mouse tumor samples (Figure 9A). Immunohistochemical analysis showed that overexpression and knockdown of *tRF-27* achieved consistent results observed in the cells (Figure 9B).

In summary, stimulated by trastuzumab, G3BPs attached to TSC complexes to LAMPs on the surface of lysosomes, thereby inhibiting MTORC1 activation.* tRF-27* overexpression blocked the lysosomal localization of TSC complexes by competitively binding with G3BPs to LAMPs. As the inhibition on MTORC1 was relieved, the growth of trastuzumab-stimulated cells was promoted, thus enhancing their resistance against trastuzumab (Figure 9C).

## Discussion

New tyrosine kinase inhibitor (TKI) drugs and antibody-drug conjugate (ADC) drugs have been combined in the treatment of HER2+ breast cancer patients 5, 47, but none can replace trastuzumab. In the present study, we analyzed the therapeutic potential of tRNA-derived fragments.

We identified *tRF-27*, which plays an important role in trastuzumab resistance, through RNA sequencing. We found that its expression level was higher in trastuzumab-resistant patients than in trastuzumab-sensitive patients, and it could be used to predict trastuzumab resistance. Cells with *tRF-27* overexpression and knockdown were used to perform cell function assays. We found that *tRF-27* overexpression enhanced cell tolerance to trastuzumab, while* tRF-27* knockdown inhibited cell tolerance. We believe that *tRF-27* can be used as a biomarker for predicting trastuzumab resistance, and it also plays an important role in the rise of trastuzumab resistance.

The function of G3BPs in trastuzumab resistance has not been studied. Our study showed that the expression of G3BPs did not change significantly during breast cancer development and trastuzumab resistance.* tRF-27* is supposed to function relying on G3BPs. Our study confirms that cancer cells become more tolerant to trastuzumab when G3BPs are silenced, suggesting that G3BPs influence trastuzumab resistance with their expression levels unchanged.

Our research also revealed a particular molecular mechanism of tRFs in cancer development. For example, RNAs can perform a specific biological function by competing with the domains of other proteins. We found that during this process, the expression of the protein may no change significantly, but its function change greatly. The NTF2 domain combined with *tRF-27* may be employed as a target for designing new treatments for trastuzumab-resistant breast cancer patients in the future.

We initially viewed* tRF-27* as a miRNA which can function through complementary pairing of certain bases. However, we found that *tRF-27* bound to G3BPs, not the proteins that we are familiar with, such as AGO2. Although stress granules can act as an alternative to preinitiation complexes (PICs) upon stress 48, we did not observe an association between *tRF-27* and stress granules. On the one hand, G3BPs have not been reported to infect miRNA; on the other hand, G3BPs cannot form stress granules under the stimulation of trastuzumab. Finally, we detected the expression of genes in the mTOR pathway (*mTOR*, *PI3K*, *PTEN*, *AKT*, *RPTOR*) with the RIP assay products of G3BPs; the results of qRT-PCR showed no binding of G3BPs to these genes. This leads us to speculate that G3BPs might act as RNA-binding proteins (RBP) to function in a novel way.

tRNA-derived fragments may function in tumor-related events through specific mechanisms. For example, *tRF-21* functions by occupying the phosphorylation site of the protein 14. In the present study, we found that *tRF-27* might employ a similar mechanism. We had thought that the expression levels of G3BPs might change during trastuzumab tolerance. However, our experimental and clinical data confirmed no significant change in the expression level of G3BPs. We believe that the expression of* tRF-27* may be affected by G3BPs, but external factors.

In previous reports, G3BPs exerted an inhibitory effect on MTORC1 in a non-redundant manner. Studies have shown that when G3BP1 is completely knocked out, overexpression of G3BP2 cannot reverse the activation of MTORC1. Researchers have focused on the function of G3BP1, rather than G3BP2. In the present study, we found that *tRF-27* bound directly to G3BP1/G3BP2 without relying on the dimers formed with G3BPs, suggesting no specific difference between the bindings to G3BP1 and G3BP2. Moreover, G3BP1 or G3BP2 exhibited a strong homology in the NTF2 domain. We believe that the binding of *tRF-27* to G3BPs is more dependent on this domain, which is in line with our experimental results. Unfortunately, our results are not sufficient enough to prove the specificity of G3BP2 in this binding. During our experiments, G3BPs performed in a similar manner, which needs to be studied further.

In our study, it was difficult to effectively and accurately quantify the binding of *tRF-27* to G3BPs in cells. We transfected *tRF-27* fragments with fluorescent tags and found extensive cytoplasmic localization of* tRF-27*. This hinders us to observe the colocalization of *tRF-27* with G3BPs by immunofluorescence. Similarly, because of the extensive cytoplasmic localization of G3BPs, it was difficult to accurately localize them to the lysosomes. Finally, in our experiments, this binding was shown indirectly associated with the colocalization of downstream Tuberin (TSC2) with lysosomes.

We predicted the structure of *tRF-27* using HDOCK. A complete* tRF-27* has a loop structure, which can be destroyed by incomplete truncation. Since *tRF-27* is derived from *tRNA-cysgca*, and tRNA has complex RNA modifications and special tertiary structures, we speculate that these characteristics inherited from tRNA may lay a foundation for the biological function of tRNA-derived fragments, which should be verified in our future research.

## Conclusions

*tRF-27-ZDXPHO53KSN* can be used as a biomarker for predicting trastuzumab resistance in HER2-positive breast cancer. The expression of *tRF-27* is associated with drug tolerance of HER2-positive breast cancer cells. *tRF-27* binds to G3BPs in cells. G3BPs are key nucleation proteins for stress granules and regulate MTORC1 activation. Trastuzumab stimulation does not promote granule formation. In contrast, *tRF-27* relies on a specific sequence to compete with LAMP1 in binding to the NTF2 domain of G3BPs. As a result, the lysosomal localization of the TSC complex is curbed, and MTORC1 is activated to promote tumor cell proliferation. Upon stimulation by trastuzumab, *tRF-27* binds to G3BPs to inhibit the lysosomal localization of TSC, thereby activating the MTORC1 pathway to promote cell proliferation and ultimately increase trastuzumab resistance.

## Supplementary Material

Supplementary figures and tables.

## Abbreviations

G3BP1/G3BP2: Ras GTPase-activating protein-binding proteins 1 and 2
tRF: tRNA-derived fragment
MTORC1: mechanistic target of rapamycin complex 1
LAMP1: lysosomal associated membrane protein 1
ADCC: antibody-dependent cell-mediated cytotoxicity
TSC: tuberous sclerosis complex
qRT-PCR: quantitative real-time PCR
IF: Immunofluorescence
IHC: Immunohistochemistry
RIP: RNA immunoprecipitation
Co-IP: co-immunoprecipitation
TCGA: The Cancer Genome Atlas
GEO: Gene Expression Omnibus
PFS: progression-free survival
CCK-8: Cell Counting Kit-8
RPTOR: regulatory associated protein of MTOR complex 1
SPAG5: sperm associated antigen 5
4EBP1: eukaryotic translation initiation factor 4E binding protein 1
TKI: Tyrosine kinase inhibitor
ADC: antibody-drug conjugate
