# Supplementary material for: tRF-27 competitively Binds to G3BPs and Activates MTORC1 to Enhance HER2 Positive Breast Cancer Trastuzumab Tolerance
Source: Int J Biol Sci. 2024 Jul 15;20(10):3923–41. doi: 10.7150/ijbs.87415 (PMC11302882; doi:10.7150/ijbs.87415)
Supplement: Supplementary file 1 — Supplementary figures and tables. [file ijbsv20p3923s1.pdf]

Supplementary File

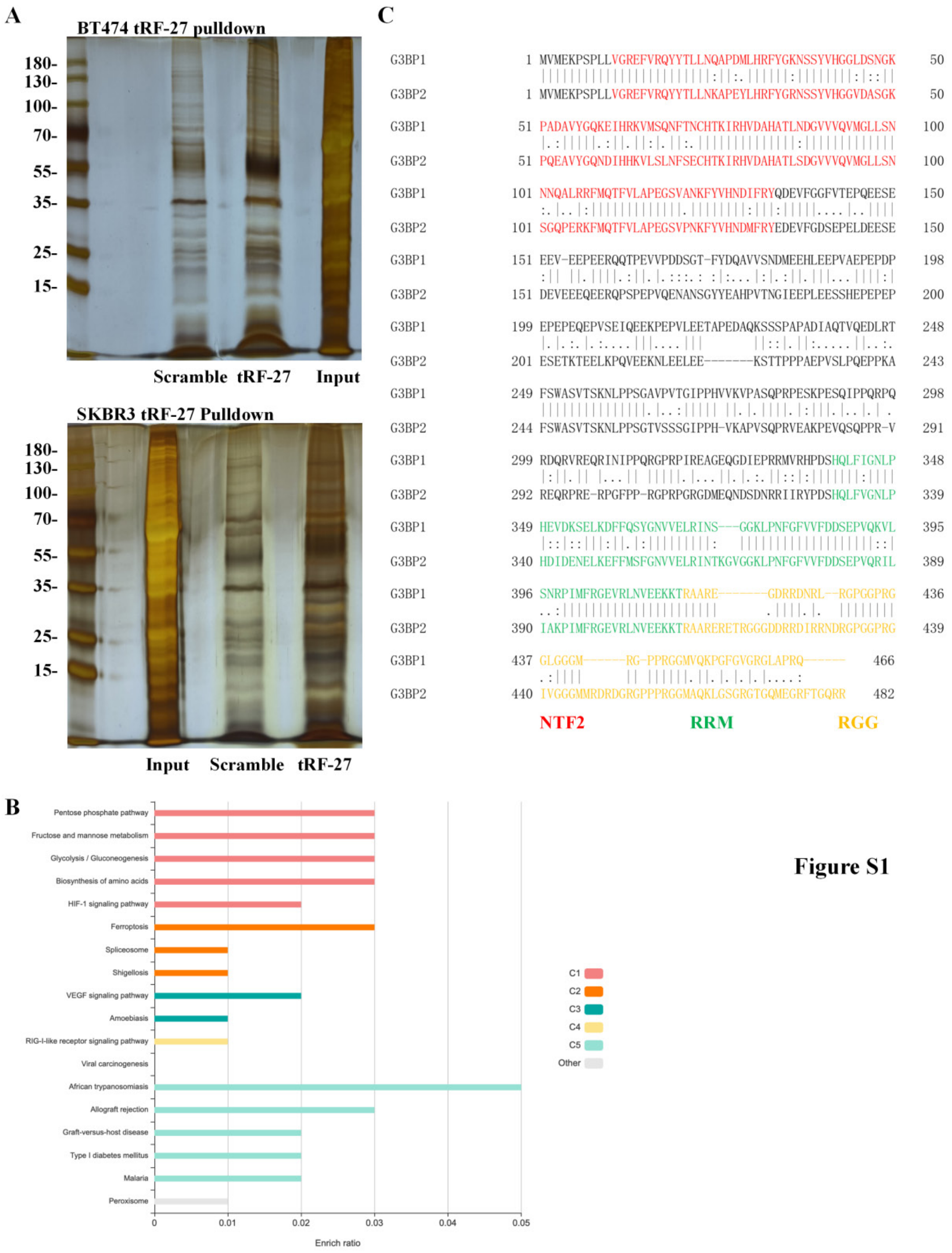

Figure S1

Figure S1: *tRF-27* pull-down assay and sequence of G3BPs

A. Silver-stained strips for the *tRF-27* pull-down assays with BT474 and SKBR3 cells. **B**. We obtained and identified proteins from the *tRF-27* pull-down assay with JIMT1, SKBR3 and BT474

cells. We performed protein enrichment analysis of mass spectrometry results in DAVID. **C**. Sequences of G3BPs. G3BP2 and G2BP1 shared 70% homologous sequences. We identified the NTF2, RRM and RGG domains of G3BPs.

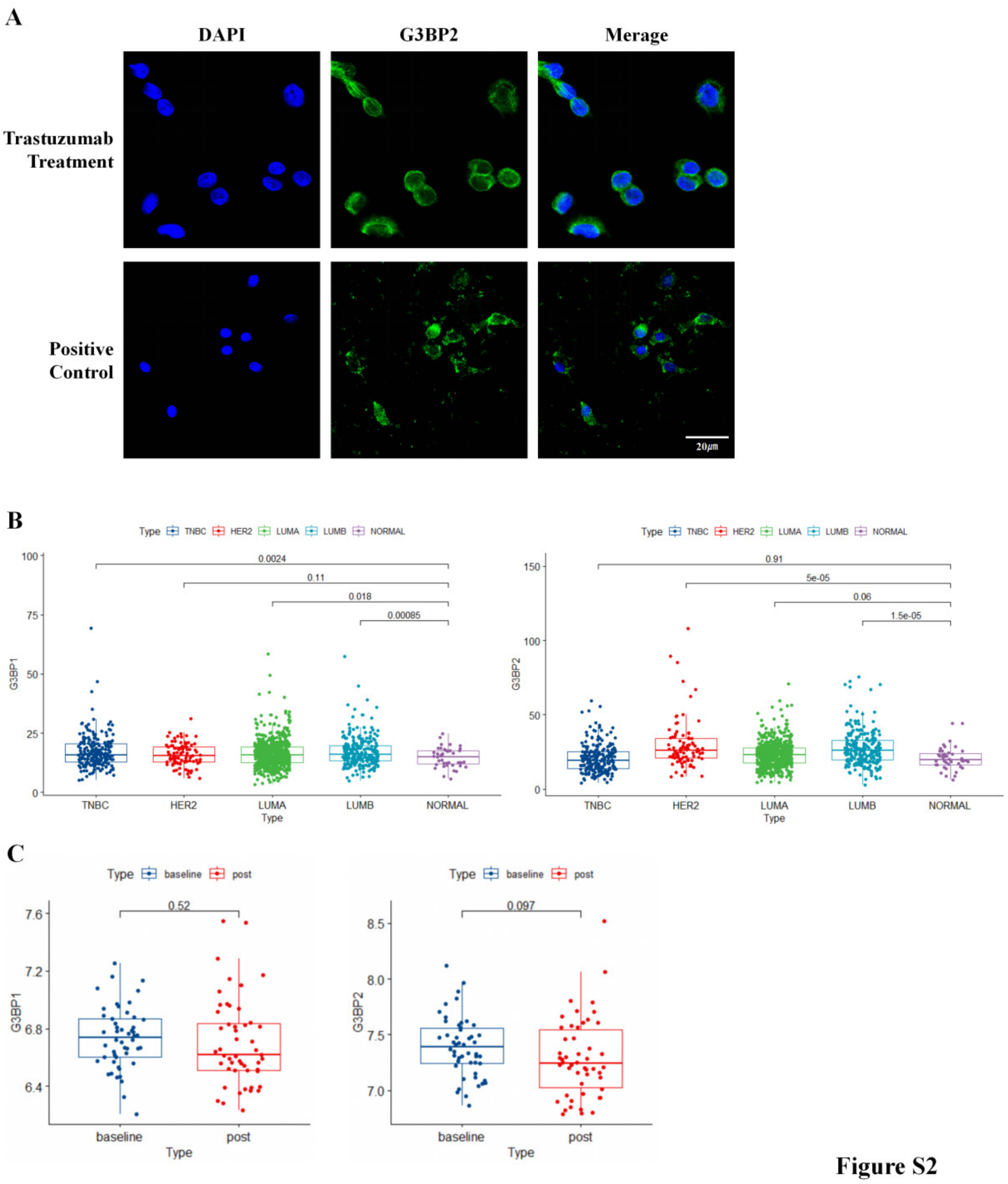

Figure S2

Figure S2: Localization of G3BPs and expression of G3BPs in HER2-positive breast cancer

A. Immunofluorescence assay of G3BP2 in JIMT1 cells; stress granules formed in a nutrient-deficient environment (Positive control), while the trastuzumab group did not show nucleated stress granules (Trastuzumab treatment). **B**. The TCGA-BRCA dataset was divided into

5 subtypes based on the PAM50 method in Genefu R package. The expression levels of *G3BP1* and *G3BP2* showed no significant differences across the relevant subtypes. **C**. GSE76360 dataset included the data before and after treatment in 50 patients receiving neoadjuvant therapy. By comparing pre- and post-treatment mRNA expression levels of *G3BP1* and *G3BP2*, we found no significant changes in *G3BP1* and *G3BP2*.

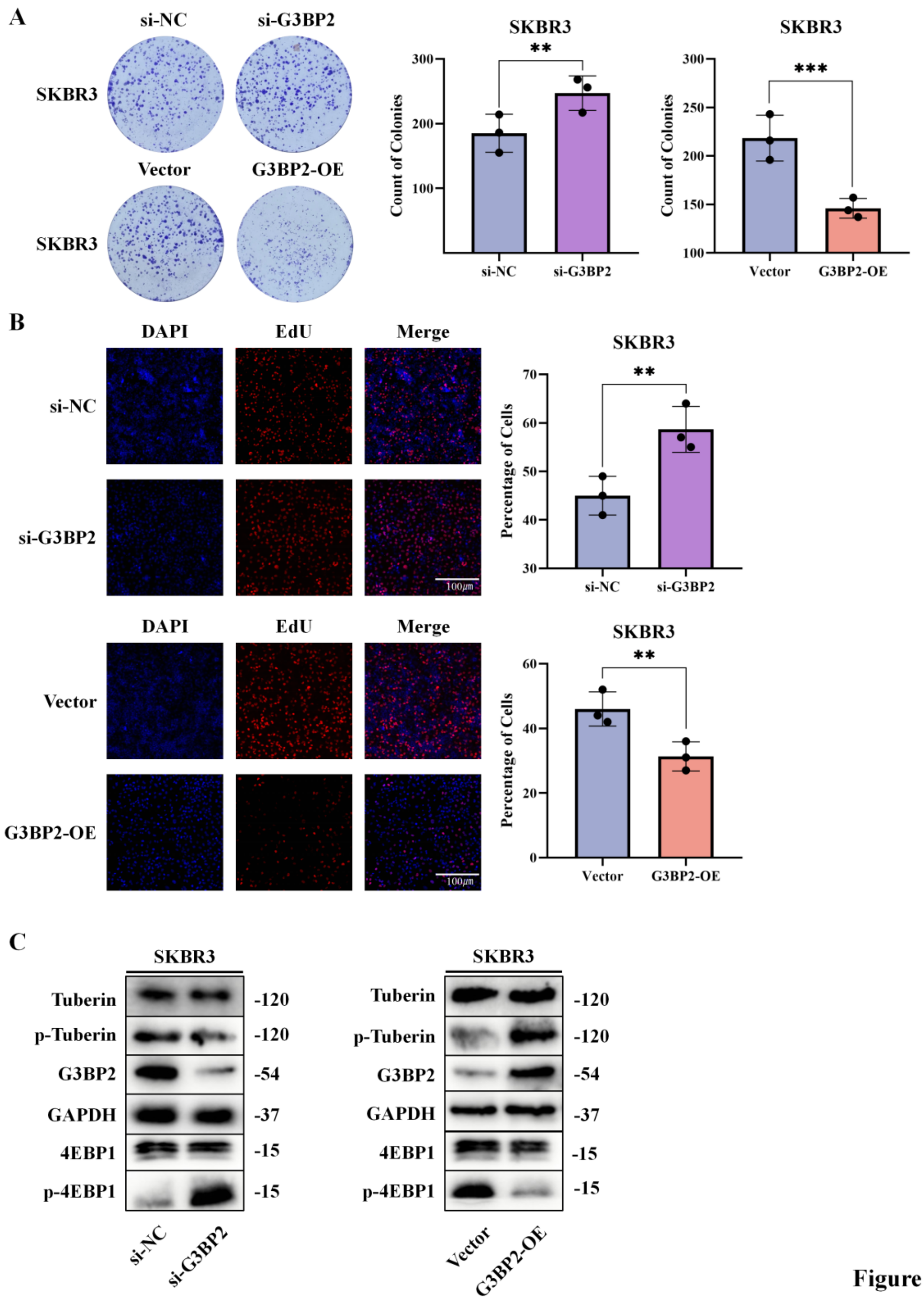

Figure S3

Figure S3: Overexpression of G3BPs weaken resistance against trastuzumab

SiRNA of G3BP2 was transfected into SKBR3 cells. Plasmids that could overexpress G3BP2 were constructed and transfected into SKBR3 cells. **A.** Cell colony formation assay was performed. The experiment was repeated for three times. Data were shown as mean ± SEM;

*P<0.05, **P<0.01, ***P<0.001. **B.** EdU assay was used to illustrate the proliferation of cells. G3BP2 knockdown promoted cell growth and proliferation. G3BP2 overexpression inhibited the growth of cells. **C**. Western blotting was used to detect protein expression in cells exposed to trastuzumab. Knocking down G3BP2 promoted the phosphorylation of Tuberin, but inhibited the phosphorylation of substrate 4EBP1 of MTORC1. Overexpression of G3BP2 inhibited the phosphorylation of Tuberin and promoted the phosphorylation of 4EBP1.

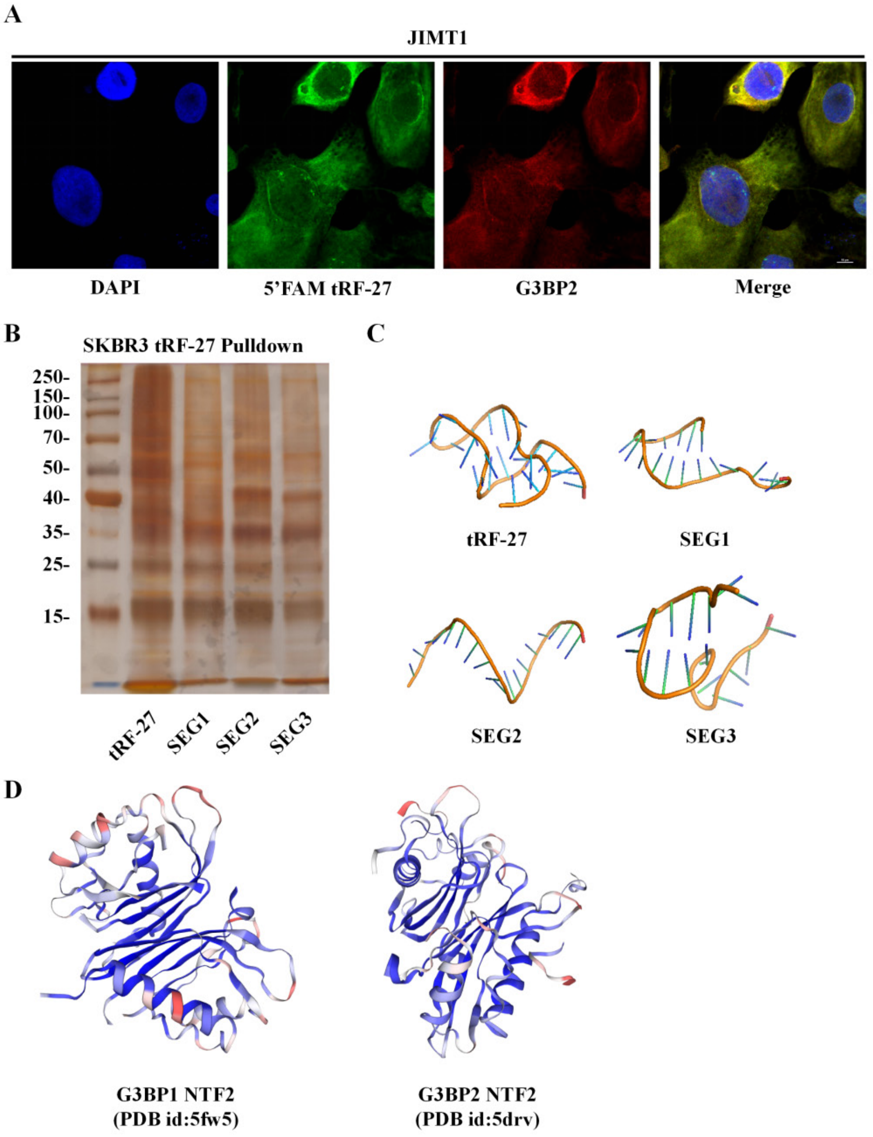

Figure S4

Figure S4: Structures of *tRF-27* and G3BPs

A. *tRF-27* with 5'-FAM was transfected into JIMT1 cells; the G3BP2 protein was immunofluorescence-stained; both were predominantly present in the cytoplasm. **B**. Silver-stained strips for RNA pull-down assay using SKBR3. **C**. Online docking tools were used to predict the

structures of the four RNAs. Complete tRF-27 had a loop, which was destroyed when tRF-27 lost the middle nine bases. **D**. NTF2 domain of G3BP1 (PDB id:5fw5) and G3BP2 (PDB id:5drv). PDB files were downloaded from Swiss-Model. **E**. The transmembrane regions of LAMP1. **F**. HDOCK was used to predict the binding of LAMP1 with the NTF2 domain in G3BPs, and the results were demonstrated with PyMol.

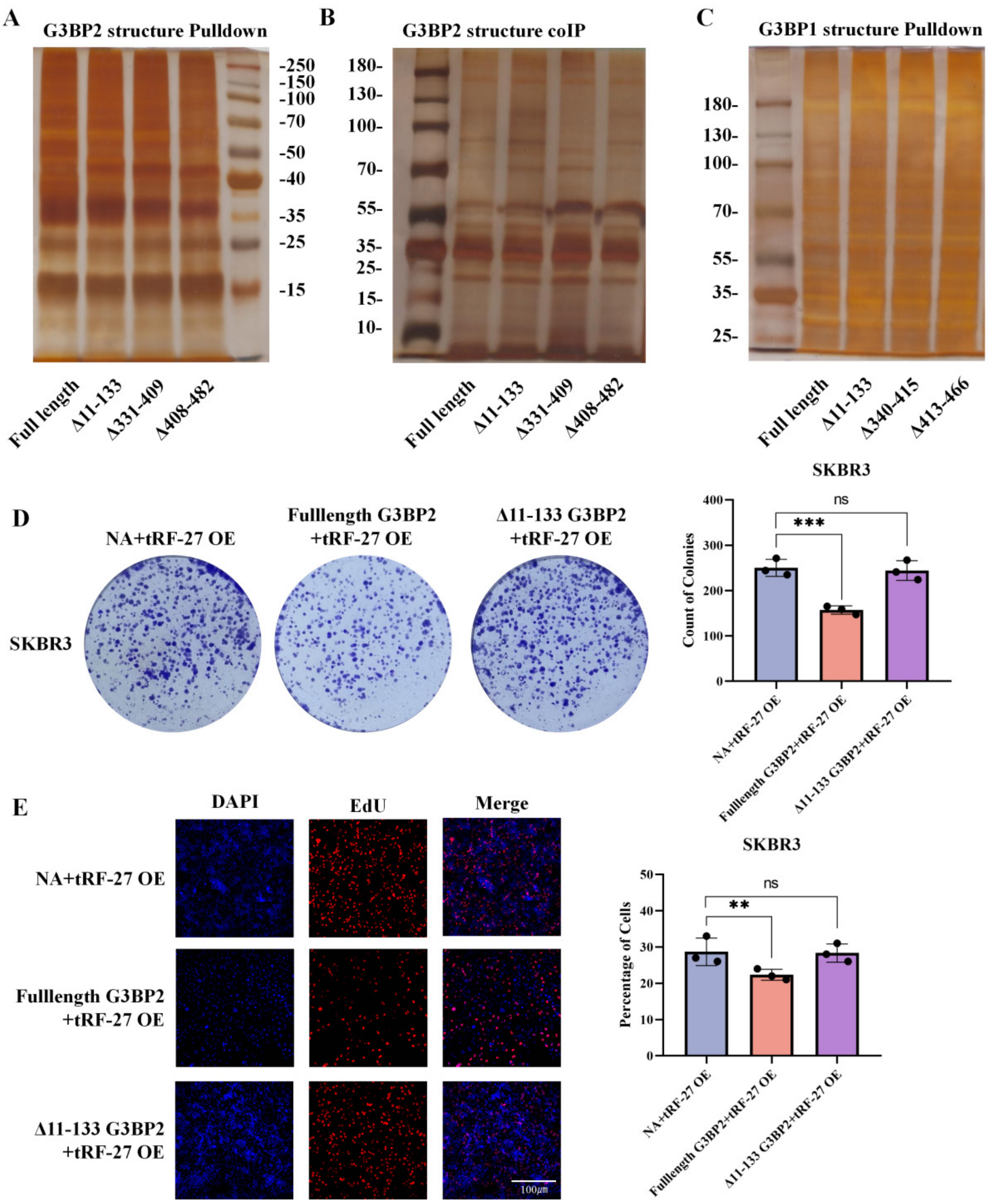

Figure S5

Figure S5: Silver-stained strips of pull-down and co-IP assays

A. Pull-down assay with the probes of *tRF-27*. The input lysates were extracted from HEK293T cells transfected with plasmids of full-length and truncated G3BP2. **B**. HA-TAG co-IP assays. The input lysates were extracted from HEK293T cells transfected with plasmids of full-length and

truncated G3BP2. **C**. Pull-down assay with the probes of *tRF-27*. The input lysates were extracted from HEK293T cells transfected with Plasmids of full-length and truncated G3BP1. **D.** G3BP2-expressing plasmids and G3BP2(△11-133) expressing plasmids were transfected into SKBR3 cells in a trastuzumab-exposed environment. Cell colony formation assay was performed. The experiment was repeated for three times. Data were shown as mean ± SEM; *P<0.05, **P<0.01, ***P<0.001. **E**. EdU assay showed the proliferation of cells in the *tRF-27* overexpression group was significantly inhibited, but not in the group overexpressing G3BP2 that lost NTF2 domain.

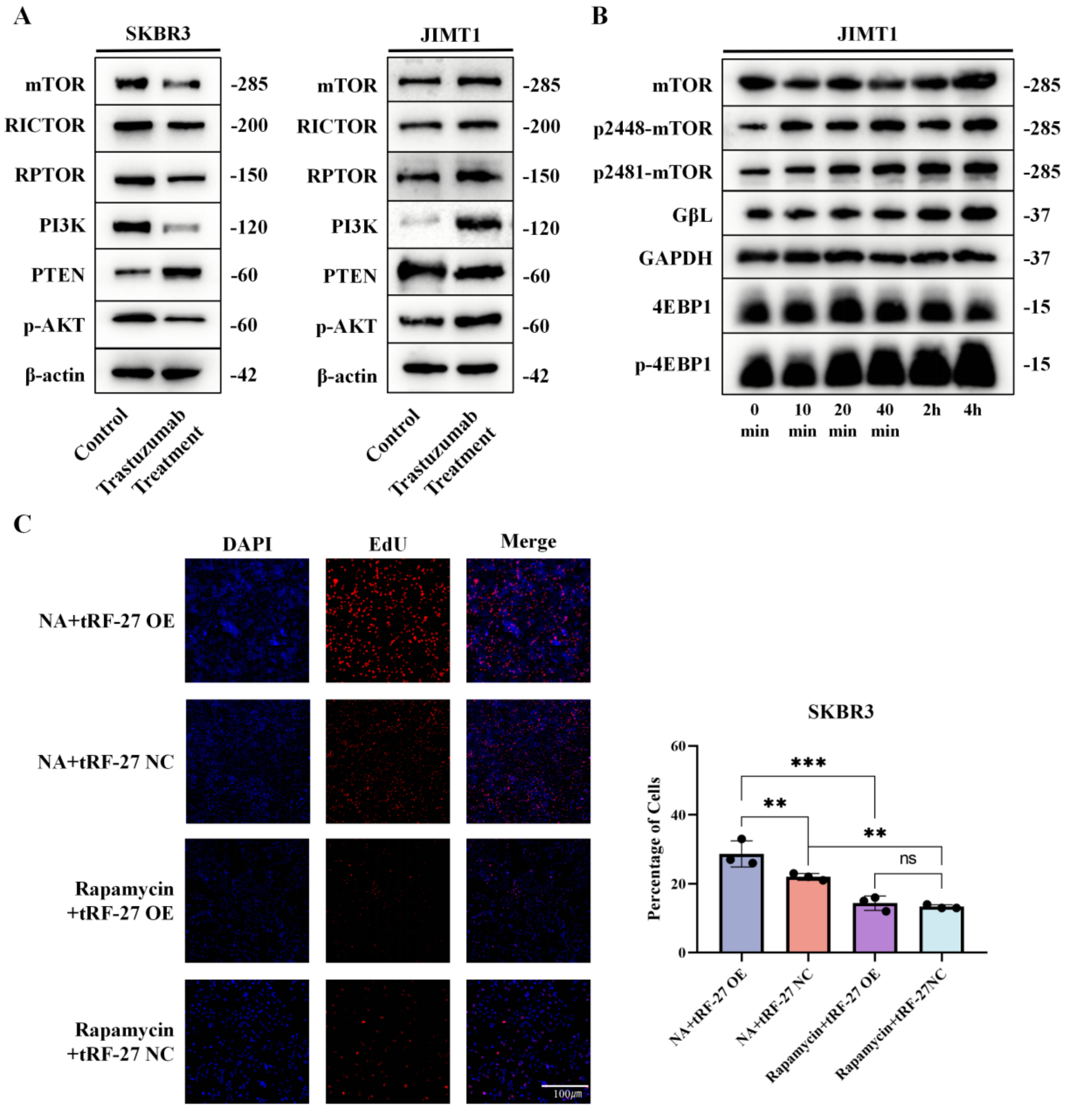

Figure S6

Figure S6: High expression of *tRF-27* blocked lysosomal localization of TSC complexes by competitively combining G3BPs with LAMPs.

A. Trastuzumab inhibited the PI3K/AKT/mTOR signaling pathway in SKBR3 cells. In trastuzumab-resistant JIMT1 cells, we observed abnormal activation of this signaling pathway. **B.**

Abnormal activation of the MTORC1 pathway. The cells were stimulated by trastuzumab for 0 min, 10 min, 20 min, 40 min, 2 h, 4 h; protein was extracted for Western blotting. **C**. Cells were treated with rapamycin, an inhibitor of MTORC1, in combination with trastuzumab, and the pro-proliferation effect of *tRF-27* on cells was largely eliminated. The experiment was repeated 3 times. Data were shown as mean ± SEM; *P<0.05, **P<0.01, ***P<0.001.

Table S1:Antibodies Used for the Research

Protein	Conpany	ID	Usage
G3BP1	CST	61559	IP,IF,WB,IHC
G3BP1	Proteintech	66486-1-Ig	IP,IF,WB,IHC
G3BP2	Abcam	ab86135	IP,IF,WB,IHC
Tuberin	Proteintech	24601-1-AP	IF,WB,IHC
p-Tuberin	Proteintech	29000-1-AP	WB,IHC
RPTOR	CST	48648	IF,WB,IHC
RPTOR	Proteintech	20984-1-AP	IF,WB,IHC
4EBP1	CST	9644	WB,IHC
p-4EBP1	CST	2855	WB,IHC
HA-TAG	CST	5017	IP,WB
HA-TAG	Proteintech	66006-2-Ig	IP,WB
SPAG5	Proteintech	14726-1-AP	IP,WB
β-actin	Abcam	ab8226	WB
GAPDH	Abcam	ab8245	WB
LAMP1	CST	9091	IF,WB
LAMP1	Proteintech	67300-1-Ig	IF,WB
mTOR	CST	2983	WB
p-mTOR	CST	5536	WB
p-mTOR	CST	2974	WB
PI3K	Abcam	ab32089	WB
AKT	Abcam	ab8805	WB
p-AKT	Abcam	ab38449	WB
PTEN	Abcam	ab267787	WB
SRC	Proteintech	11097-1-AP	WB
GβL	CST	3274	WB
S6K1	Proteintech	ab32529	WB
P-S6K1	Proteintech	ab59208	WB
P-PTEN	Abcam	109454	WB

Table S2:MS Results of TRF-27 Pulldown Assays

127 elements included exclusively in "List 1":	128 elements included exclusively in "List 1":	143 elements included exclusively in "List 1":
**BT474**	**SKBR3**	**JIMT1**
KV230	KLF6	ZN236
H3C	TYY1	ZCCHV
H2B2E	TRY6	ZBED6
H12	RUXG	XP32
RL26	RSMN	UTP15
CD11A	IGLC3	UT14A
ATD3C	IF4A1	UBP2L
FILA	HGB1A	UACA
VWA8	ZN768	TTL11
CC88B	ESRP1	TRIM4
DISP2	ACINU	TRI25
ISM1	ARF4	TLL1
EXOC5	CATIN	TLE7
P4HA2	CCAR1	TKTL1
NEBL	CDK12	TIGD6
MYO1D	CISD3	TIF1B
**FA9**	**CNBP**	**TFG**
CO3	COF2	TCPW
**FIBA**	**COPA**	**TCPH**
REL1	COPB	STAG3
B3A2	CRCDL	SPT21
IC1	CRIP1	SPB1
S10A9	CRIP2	SPAT7
S10A6	CTCF	SM34A
NFL	CYC	SERA
DPOLA	DAAF1	SENP7

CFC1	DHX8	SEC13
PLAK	DHX9	SC16A
H15	DUS1L	SAC31
**ITB6**	**EFTU**	**RTCB**
**TFEB**	**ERH**	**RS7**
NEBU	ESRP2	RS15
HXA7	EWS	RS12
RECQ1	F120A	RS10L
LEG7	FAS	RS10
SRP09	FBRL	RNPC3
DPOG1	FIZ1	RLA2
RRP1	G3BP2	RL5
FOXL2	GSDMC	RL31
RBM3	H2A1C	RL10A
TNAP2	H33	RED2
KPCT	HNRPF	RBP56
ELP6	HYALP	RBM14
TP53B	IDHP	RBBP6
DMP1	IF4A2	RADI
GRB10	IF4B	PUR6
PTPRS	IGHG1	PRKDC
CDSN	IGLC1	PRDX6
DPYL2	IGLC2	PPIB
SYPL1	ITCH	PKP3
SWAHA	K1C17	PKP2
ASTRB	K1C18	PCDB3
EIPR1	LASP1	PCBP3
HERC4	LUC7L	PCBP2
GPTC4	MAP2	PCBP1
PRA20	MAP9	PARN
2ABD	MAS1L	PABP4

FAAH2	NAL10	P5CR3
LAIR1	NHS	OTUD4
THOC7	NYNRI	OTUB1
**RSLBA**	**PCKGM**	**OTOF**
UD2A3	PGK2	NED4L
**ZN493**	**PININ**	**NEBU**
DHX29	PKP3	NCPR
PODN	PR38B	MYO5B
RGPS2	PRDX2	MYO1G
SAM9L	PRP4B	MYL6B
BRSK2	PSMD3	MYL6
CC022	RBM25	MYH9
IQUB	REPI1	MYH1
ABCF1	RL18A	MTHFS
INADL	RL5	MOES
PUM2	RL6	MOB1A
NDNF	RL8	MCM7
RBCC1	RNPS1	MAGA5
NUDC2	RS2	MA7D1
STON2	RS27L	LSM12
TITIN	RS4Y2	LMOD1
CTTB2	RSMB	LCN1
ASM3B	RU17	LC1L1
RSLAA	RU1C	KVD24
RBP56	RU2A	KV224
ISOC2	RU2B	KIF2C
ZG16B	RUXE	KCMF1
S10AG	RUXF	KAT1
PRR11	RUXGL	IZUM4
SPTCS	S39A7	ITCH
SRAC1	SAP18	IST1

SPB12	SCNBA	IKZF3
SERC2	SLMAP	IFT74
PI3R4	SMC3	IFIT1
KRA94	SMD3	HSPB8
GRB1L	SNRPA	HSP77
DHX36	SP1	HSP76
RC3H2	SP3	HNRPU
TF7L2	SPIB	HNRPK
SDA1	SPTB2	HNRPC
ARGL1	SR140	HBD
PAQR5	SRS10	H31
SPAT7	SRS11	H2AY
CFDP1	SRSF1	H2AX
NPCL1	SRSF3	GRSF1
PKHG1	SRSF5	GDIB
G3BP2	SRSF6	G3BP2
ANR26	SWAHA	FUS
SRRM2	TACC1	FLNA
ASTER	TBA3D	FAS
MEMO1	TBA4A	EZRI
TBB8	TBB8	ESRP1
TBB2A	TIF1A	EPN4
SRSF4	TIF1B	ELP6
KV229	TOP1	EHD3
H2B1O	TRAF2	EHD1
H2AJ	TRIM3	EF1D
H13	TRY1	DUX4C
AMACR	TRY2	DUS21
TBB2B	TV23C	DP13A
KVD28	TYY2	DHX36
H2B1B	U2AF2	DHB12

H14	ZC3HD	44896
ADDB	ZFP91	DDX3Y
RL26L	ZN316	DDX3X
NAC1	ZN577	CT2NL
HSP72	ZN638	CRYAB
CD11B	ZN649	COPB
ATD3B	ZN672	CI114
	ZNF8	CFA91
		CAZA2
		CAZA1
		CAPR1
		CALB1
		ARF4
		AP2M1
		AP1S1
		AKA12
		AHNK
		ACTN4
		ACTBL
		ACL6A
		A16A1
		2AAA
		1433T
